# Neonatal gut and respiratory microbiota: coordinated development through time and space

**DOI:** 10.1186/s40168-018-0566-5

**Published:** 2018-10-26

**Authors:** Alex Grier, Andrew McDavid, Bokai Wang, Xing Qiu, James Java, Sanjukta Bandyopadhyay, Hongmei Yang, Jeanne Holden-Wiltse, Haeja A Kessler, Ann L Gill, Heidie Huyck, Ann R Falsey, David J Topham, Kristin M Scheible, Mary T Caserta, Gloria S Pryhuber, Steven R Gill

**Affiliations:** 1 Genomics Research Center, University of Rochester School of Medicine and Dentistry, Rochester, NY USA; 2 Department of Biostatistics and Computational Biology, University of Rochester School of Medicine and Dentistry, Rochester, NY USA; 3 Department of Microbiology and Immunology, University of Rochester School of Medicine and Dentistry, 601 Elmwood Avenue, Rochester, NY 14642 USA; 4 Medicine-Infectious Disease, University of Rochester School of Medicine and Dentistry, Rochester, NY USA; 5 Center for Vaccine Biology and Immunology, University of Rochester School of Medicine and Dentistry, Rochester, NY USA; 6 Division of Neonatology, Department of Pediatrics, University of Rochester School of Medicine and Dentistry, Rochester, NY USA; 7 Division of Infectious Disease, Department of Pediatrics, University of Rochester School of Medicine and Dentistry, Rochester, NY USA

## Abstract

**Background:**

Postnatal development of early life microbiota influences immunity, metabolism, neurodevelopment, and infant health. Microbiome development occurs at multiple body sites, with distinct community compositions and functions. Associations between microbiota at multiple sites represent an unexplored influence on the infant microbiome. Here, we examined co-occurrence patterns of gut and respiratory microbiota in pre- and full-term infants over the first year of life, a period critical to neonatal development.

**Results:**

Gut and respiratory microbiota collected as longitudinal rectal, throat, and nasal samples from 38 pre-term and 44 full-term infants were first clustered into community state types (CSTs) on the basis of their compositional profiles. Multiple methods were used to relate the occurrence of CSTs to temporal microbiota development and measures of infant maturity, including gestational age (GA) at birth, week of life (WOL), and post-menstrual age (PMA). Manifestation of CSTs followed one of three patterns with respect to infant maturity: (1) *chronological*, with CST occurrence frequency solely a function of post-natal age (WOL), (2) *idiosyncratic to maturity at birth*, with the interval of CST occurrence dependent on infant post-natal age but the frequency of occurrence dependent on GA at birth, and (3) *convergent*, in which CSTs appear first in infants of greater maturity at birth, with occurrence frequency in pre-terms converging after a post-natal interval proportional to pre-maturity. The composition of CSTs was highly dissimilar between different body sites, but the CST of any one body site was highly predictive of the CSTs at other body sites. There were significant associations between the abundance of individual taxa at each body site and the CSTs of the other body sites, which persisted after stringent control for the non-linear effects of infant maturity. Canonical correlations exist between the microbiota composition at each pair of body sites, with the strongest correlations between proximal locations.

**Conclusion:**

These findings suggest that early microbiota is shaped by neonatal innate and adaptive developmental responses. Temporal progression of CST occurrence is influenced by infant maturity at birth and post-natal age. Significant associations of microbiota across body sites reveal distal connections and coordinated development of the infant microbial ecosystem.

**Electronic supplementary material:**

The online version of this article (10.1186/s40168-018-0566-5) contains supplementary material, which is available to authorized users.

## Background

Human life is dependent on a diverse community of symbiotic microbiota that have co-evolved with their human hosts to modulate crucial aspects of normal physiology, metabolism, immunity, and neurologic function [1]. Our relationships with microbes begin in utero. Limited microbial communities observed immediately after birth expand into densely colonized, diverse bacterial ecosystems within the first weeks of life. Early interactions that occur between members of the microbial community and between microbes and their human host are responsible for features of postnatal development that influence future health [2–5]. The newborn infant microbiota is highly dynamic and undergoes rapid changes in composition through the first years of life towards a stable adult-like structure with distinct microbial communities of unique composition and functions at specific body sites [5–10]. Relatively little has been reported about longitudinal microbiota development or compositional differentiation across multiple body sites during this period. This is particularly true for high-risk pre-term infants, who because of immature mucosal and skin barriers, as well as underdeveloped immunity and suboptimal nutrition, are at increased risk for invasive infection and dysregulated inflammation of critical systems, namely the respiratory and gastrointestinal tracts. Serious perinatal complications in these pre-term infants result in prolonged hospitalization, treatment with antibiotics, and delays in enteral feeding that influence interactions with microbes and inhibit microbial colonization characteristic of full-term infants [11].

While numerous microbial communities within individual body sites have been described [3, 9, 11–14], associations between the microbiota across multiple body sites or systems are less well studied [11, 15, 16]. A better understanding of the microbiota landscape and interactions across multiple body sites is needed to assess the influence of perturbations in one system on the microbiota of other systems. Elucidating the direct and indirect interactions of microbiota across multiple body sites presents a formidable analytical challenge. Available statistical methods vary widely in sensitivity and precision, with no consensus on the best approach [17]. Community profile data from 16S rRNA amplicon surveys is compositional, high-dimensional, and generally observational. Limited validation of these interactions through independent experiments or modeling leaves researchers without the data needed to reconstruct authentic interaction networks and to make meaningful biological conclusions [18]. The limited body of literature reporting on cross-body site interactions is a testament to these challenges [11, 15, 16]. Our study leverages dimension reduction and longitudinal modeling techniques, allowing for the effects of within body site temporal development and cross-body site associations during early life to be distinguished and quantified. Furthermore, unlike previous studies that sampled the microbiome parsimoniously across body sites, our study sampled multiple body sites from a large cohort of pre- and full-term infants at frequent and regular intervals throughout their first year of life, within the crucial window of time when the microbial community maximally influences immune development and potential long-term health outcomes, including atopy, inflammatory bowel diseases, and subtleties of neurodevelopment [19–22].

Here, we describe and compare patterns of development of the microbiota of the nose, throat, and gut over the first year of life in 82 pre- and full-term infants (Table 1). Within the three body sites, we characterized development as a pattern of progression through microbiota community state types (CSTs), each differentiated by the abundance of specific taxa. Classifying samples into CSTs based on composition provides a useful summary of the state of the microbial community within a particular body site at a given time. The longitudinal pattern of CST occurrence represents a conceptually and analytically tractable summary narrative of microbiota development, with occurrence patterns of individual CSTs reflecting facets of developmental phenotype. Leveraging this framework of CSTs and their occurrence patterns as a proxy for high level characteristics of microbiota development, we compared full- and pre-term infants on the basis of their progression through these CSTs and assessed the associations between CST prevalence, gestational age (GA) at birth, post-natal age as measured by week of life (WOL), and developmental age as indicated by post-menstrual age (PMA: equal to GA plus WOL). While the occurrence of all CSTs was associated with time, each CST exhibited one of three distinct temporal patterns which varied in the extent to which GA at birth, WOL, and PMA influenced CST manifestation. First, a *chronological* pattern in which CST occurrence was independent of GA at birth and PMA, but was instead a function of WOL, with a given CST of this type occurring at a consistent post-natal interval with similar frequency in both pre- and full-term subjects. Second, a pattern *idiosyncratic to gestational age at birth*: CSTs occurred during characteristic post-natal intervals, but their frequency was a function of maturity (GA) at birth. These CSTs are over- or under-represented in pre-term subjects. Third, a *convergent* pattern whereby lower GA at birth typically imposed a delay on the manifestation of a CST, with CST occurrence frequency in pre-term infants reaching parity with full-term infants after a post-natal interval proportional to prematurity. While a narrative of typical infant microbiota development in terms of progression through archetypal community states emerges from these observations, the distinct types of associations between CST occurrence patterns and time reveal that prematurity influences different aspects of microbiota development in unique ways, such as delaying some components of the developmental phenotype, permanently altering others, and having no discernable influence on others still. We demonstrate that although community composition is dissimilar between distal body sites, the abundance of various taxa and the occurrence patterns of CSTs are correlated across body sites. These associations cannot be entirely accounted for by the common influence of developmental or post-natal age on all body sites nor by the direct transmission of bacteria between body sites, which suggest the existence of relationships between infant development and the microbiota across body sites that have yet to be defined.

**Table 1:** Demographics and clinical variables

Variables	Pre-term (*N* = 38)	Full-term (*N* = 44)	*p* value
Gestational age at birth (weeks), *mean ± SD*	29.56 ± 3.4	39.61 ± 1.16	< 0.001
Gestational age at birth, *N*			
23–25 weeks	8	–	
26–27 weeks	6	–	
28–29 weeks	5	–	
30–31 weeks	9	–	
32–33 weeks	6	–	
34–35 weeks	4	–	
Full term	–	44	
Birth weight (kg), *mean ± SD*	1.38 ± 0.62	3.53 ± 0.56	< 0.001
Sex - male, *N %*	21 (55.3%)	27 (61.4%)	0.738
Race - Caucasian/AA/other*, *N %*	22/10/6 (57.9/26.3/15.9%)	30/5/9 (68.2/5/9%)	0.377
Ethnicity - Hispanic or Latino**, N %	4 (10.5%)	8 (20%)	0.349
Delivery method - C-section, *N %*	24 (63.2%)	21 (47.7%)	0.239
Hospital samples, collected (analyzed), *N*			
Rectal	294 (252)	43 (27)	
Nasal	288 (243)	44 (19)	
Throat	174 (159)	26 (13)	
Post-discharge samples, collected (analyzed), *N*			
Rectal	346 (331)	479 (469)	
Nasal	350 (318)	483 (433)	
Throat	202 (184)	206 (182)	
Total acute respiratory visits***, *N*	51	51	
Number of infants had acute respiratory visit***, *N*	20	24	

*Race group “Other” includes those not reported race

**Ethnicity is unknown for four full-terms

***Samples collected during monthly visits where evidence of acute respiratory infection was observed were excluded in this analysis

Overall, our results illustrate fundamental interactions between the gut and respiratory microbiomes in pre-term and full-term infants and elucidate the dichotomy between innate host developmental programming driven solely by post-menstrual age and adaptive host developmental processes driven by post-natal environmental exposures in terms of their influence on microbiota development. Thus, this study will guide the development of criteria for therapeutic approaches that promote the establishment of a homeostatic microbiome during the critical period of neonatal development.

## Results

### Overview of infant cohort

To characterize the development of the neonatal gut and respiratory tract microbiota, we collected rectal, nasal, and throat swabs from 82 pre- and full-term infants over the first year of life (Table 1). From the 38 pre-term infants, weekly samples were collected while hospitalized in the neonatal intensive care unit from birth until discharge, and monthly samples were collected from discharge through one year of gestationally corrected age. From the 44 full-term infants in the cohort, monthly samples were collected through the first year of life, starting at birth. Samples collected during monthly visits in which evidence of acute respiratory illness was observed were excluded from this analysis (as described in the “Methods” section). The nasal swabs were selected as the primary measure of the respiratory microbiota, with throat swabs as a secondary supplemental indicator. Because the focus of this study is on microbiota development across multiple body sites, we assayed throat samples from an unbiased random subset of 40 matched subjects distributed evenly between the pre- and full-term cohorts. Microbiota from 1079 gut, 1013 nasal, and 538 throat samples were characterized by 16S rRNA amplicon sequencing.

### Microbiome community state types (CSTs) summarize archetypal states and developmental narrative

**Fig. 1:**
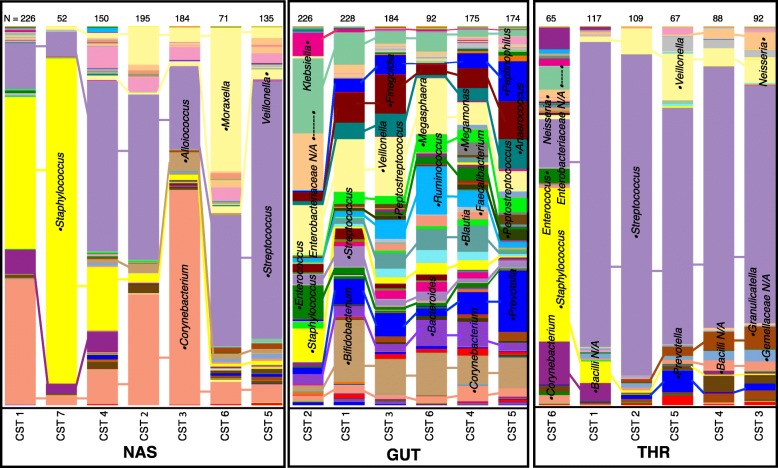
Composition of community state types (CSTs) of the nose (NAS), gut (GUT), and throat (THR). Average composition of each CST was identified by Dirichlet-Multinomial mixture (DMM) model-based clustering. Samples are grouped by the Dirichlet component that they represent, with each component corresponding to a CST, and the average composition of all samples in each CST group is represented. The CSTs in each site are ordered based on their occurrence over time (e.g., CST 2 is the earliest gut CST). The height of each bar is equal, indicating that all total abundances are normalized to a constant sum. The number of samples in each CST is included at the top of each bar. Within each bar, different colored bands correspond to different taxa, and the height of a given band is proportional to the average relative abundance of the corresponding taxon in the given CST. The top ten most abundant taxa within each body site are identified, with the closed circle flanking each taxa name positioned in the corresponding taxa in each bar. The composition of all samples is listed in Additional file 4: Table S1

**Fig. 2:**
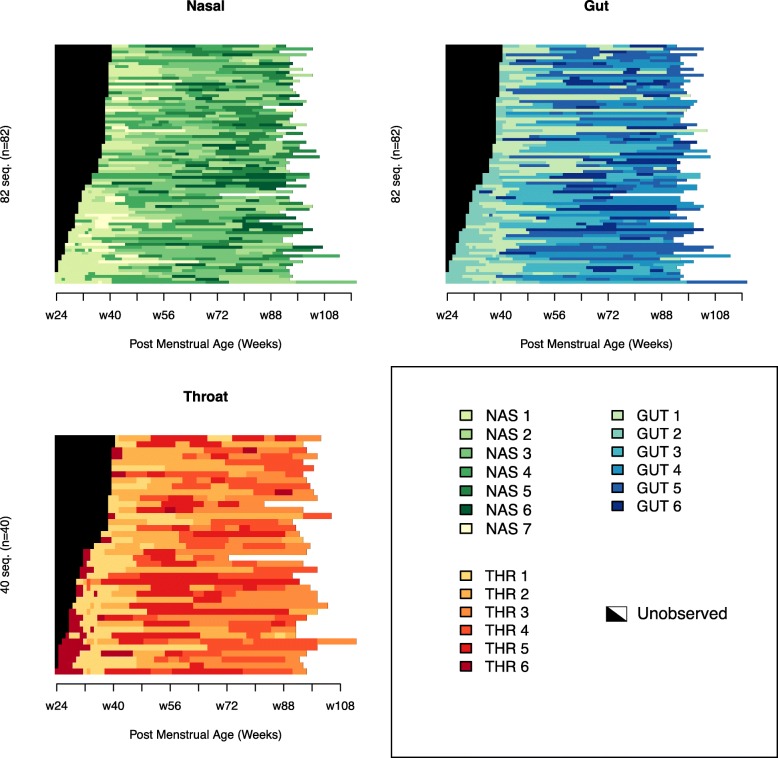
Sequence index plots indicate the progression of community state types (CSTs) over time for each subject. Subjects are stratified along the y-axis and sorted in descending order by gestational age at birth. Post-menstrual age (PMA) in weeks is indicated along the x-axis. The period of sampling for each individual is colored, with colors indicating the observed CST in a given time period. The time point of each observation is rounded down to the week in which the sample was taken, and the surrounding period of time is colored according to the CST of the sample, with color changes occurring at the midpoint between consecutive samples in which different CSTs were observed. For each subject, the black region on the left indicates the period prior to birth and the white region on the right indicates the period after the last sample was taken. In all three body sites, strong temporal structure and ordered patterns of CST progression are evident. For example, CSTs 1, 2, and 6 are overrepresented during the period prior to 40 weeks PMA in the nose, gut, and throat, respectively

The microbiota community composition of the rectal, nasal, and throat samples was quantified by 16S rRNA amplicon sequencing. To develop tractable summary representations of typical compositional profiles, samples from each body site were independently clustered into community state types (CSTs) using Dirichlet-Multinomial mixture (DMM) models [23]. The DMM model sought to explain the operational taxonomic unit (OTU) compositional vector as a sample from a mixture of different canonical Dirichlet components. For each sample, the DMM model posterior probabilities indicated which Dirichlet component the observed vector of OTU counts most likely represented. On the basis of these probabilities, samples were assigned to clusters corresponding to CSTs which collapse the variation in microbiota composition into commonly observed archetypal states that serve as summary representations of the microbiota composition at each site (Additional file 1: Figure S1). The CST of a particular sample indicates the approximate abundance of characteristic taxa and the presence of distinct motifs in the overall community composition profile (Fig. 1 and Additional file 2: Figure S2). A series of community states observed longitudinally within a given infant therefore summarizes the microbial community development in that infant over the course of the study. Additionally, occurrence pattern of CSTs can be associated with a variety of covariates, reflecting properties of the overall phenotype of microbiota development. These attributes of the CST-based approach provide an attractive framework for summarizing and characterizing infant microbiota development and for interrogating and elucidating the relationships between phenotypes of development and components of host maturity. A robust resampling procedure (see the “Methods” section) identified 6 CSTs within the gut, 7 within the nose, and 6 within the throat (Additional file 3: Figure S3), with each CST distinguished by the variance (Additional file 2: Figure S2) and relative abundance of specific OTUs (Fig. 1). The number assigned to each CST indicates the overall frequency of occurrence at each respective site, with CST 1 being the most frequent. Based on OTU abundance and the sequence of progression of CSTs over time observed in each subject (Fig. 2), we concluded that the CSTs consistently exhibited three properties: (1) they had highly dissimilar composition between different body sites, (2) they were associated with post-menstrual age (PMA), gestational age (GA) at birth, and/or week of life (WOL), and (3) they demonstrated patterns of co-occurrence such that the observation of a specific CST at a given body site was predictive of contemporaneous CSTs at other body sites.

### Microbiota composition of community state types and occurrence through developmental time

The CSTs at all three body sites displayed a range of diversity and abundance of specific OTUs (Additional file 2: Figure S2, Additional file 4: Table S1). The six throat CSTs were the least diverse, with types 1 through 5 dominated by *Streptococcus* (62–85%) and type 6 by *Staphylococcus* (41%). The seven nasal CSTs were substantially more diverse, with *Streptococcus* and *Corynebacterium* ranging from 5% to greater than 50% abundance across all CSTs. The six CSTs of the gut were the most diverse of all three body sites and were consistently populated with *Enterobacteriaceae*, *Veillonella*, *Ruminococcus*, *Streptococcus*, *Prevotella*, *Bacteroides*, and *Bifidobacterium* at mean relative abundances greater than 1%. Patterns of temporal CST progression are shared across individuals, with the typical order of gut progression as CST 2,1,3,6,4,5; nasal progression as CST 1,7,4,2,3,6,5; and throat progression as CST 6,1,2,5,4,3 (Fig. 1). The majority of infants manifest most CSTs in their first year, in a similar sequence and at similar ages (Fig. 2, Additional file 5: Figure S4). While no single CST is observed in all 82 infants, common patterns of sequential CST occurrence at each body site reveal canonical temporal orderings and a tendency towards monotonic CST progression over time. Furthermore, CSTs occurring later in this temporal progression comprised relatively more diverse microbial communities within each body site. Pre- and full-term infants are initially colonized by distinct CSTs, with CSTs 1, 2, and 6 overrepresented prior to 40 weeks PMA in the nose, gut, and throat, respectively. Temporal progression after 50 weeks PMA consists of similar CSTs in both pre- and full-term infants as they mature beyond 90 weeks PMA. Notably, individual infants matched by PMA transition through CSTs at different rates, which suggests additional factors influence microbiota progression.

In all three body sites, the CSTs most frequently observed at the earliest PMA in both pre-term and full-term infants contained high levels of *Staphylococcus*, which decreased as the infants matured beyond 39 weeks PMA. This initial transient colonization of infant gut and respiratory microbiota by *Staphylococcus* has been described in other studies and suggests that this bacterium confers early developmental metabolic and immune benefits to the host [24, 25]. Continued temporal developmental progression likely reflects adaptation of the microbiota to corresponding functional changes in the infant host, including the transition of the gut microbiota from an aerobic community in early PMA to an anaerobic community with increasing abundance of *Ruminococcus*, *Prevotella*, *Bacteroides*, and other anaerobes beyond PMA week 46. The throat and nasal sites maintain an aerobic microbiota throughout early life, dominated after week 40 PMA by *Streptococcus*, with emergence and stable colonization of *Corynebacterium*, *Alloiococcus*, *Moraxella*, and *Veillonella* in the nasal sites and *Veillonella* and *Neisseria* in the throat. Although *Moraxella* is an opportunistic respiratory pathogen, observations from our asymptomatic infant cohort are similar to previous studies which demonstrate stable colonization of *Moraxella* and *Alloiococcus* in the nasopharynx of healthy infants [26].

### Predicted functions of community state types

To identify microbiota functions over the first year of life, the functional potential of all rectal, nasal, and throat CSTs was predicted using PICRUSt [27], which infers the functional metagenome of microbial communities based on marker gene data and reference bacterial genomes. The top eight KEGG pathways with positive or negative enrichment in each CST (*p* value < 0.001, FDR < 10%, and linear discriminate analysis (LDA) score > 3.0) were used for evaluation of functional differences within all CSTs (Additional file 6: Figure S5). The initial CSTs in all three sites (nasal CSTs 1 and 7, throat CST 6, and gut CST 2), clustered into a functionally distinct group enriched in multiple pathways, including metabolism of lipids, purines, and pyrimidines. Synthesis of nucleotides from purines and pyrimidines in early neonates supports chromosomal replication and active microbiota growth essential for early colonization of epithelial surfaces [28, 29]. Energy metabolism in early (nasal CST 7, throat CST 6, and gut CST 2) and late (nasal CST 6, gut CST 1, and gut CST 5) CSTs was driven by the pentose phosphate pathway (PPP) with the production of NADPH used for anabolic reactions needed for synthesis of cellular molecules. As the infant matured and gained exposure to additional dietary substrates, there was an enrichment of genes for carbohydrate uptake (PTS), central carbon metabolism (glycolysis and pyruvate synthesis), and the TCA cycle with increased production of ATP as an energy source for microbiota in gut CSTs 1,3,4,5; nasal CSTS 2,3,6; and throat CST 3,4,5. The enrichment of two-component signal transduction (TCS) pathways following temporal progression from the cluster of initial CSTs (nasal CSTs 1 and 7, throat CST 6, and gut CST 2) to all subsequent CSTs suggested increases in communication within the microbiota community and between microbiota and host as a result of changes in the immediate host environment and availability of metabolites [30]. We next compared enriched functions in the initial (gut CST 2 and nasal CST 1) and later (gut CSTs 3,4,6 and nasal CSTs 2,3,6) nasal and gut CSTs to identify potential microbiota functions that distinguish these sites. Aside from a limited number of distinct pathways in gut CST 1, such as bisphenol degradation, the rectal and nasal microbiota share many functions at this level of pathway resolution. Taken together, our results suggest that similar to the longitudinal progression of CSTs, specific functional pathways emerge in the initial early life CSTs, with expansion and diversification of microbial communities in later CSTs occurring as a result of contact with environmental sources and adaptation to changes in energy substrates. 

### Correlations between community state type and PMA in pre- and full-term infants

**Fig. 3:**
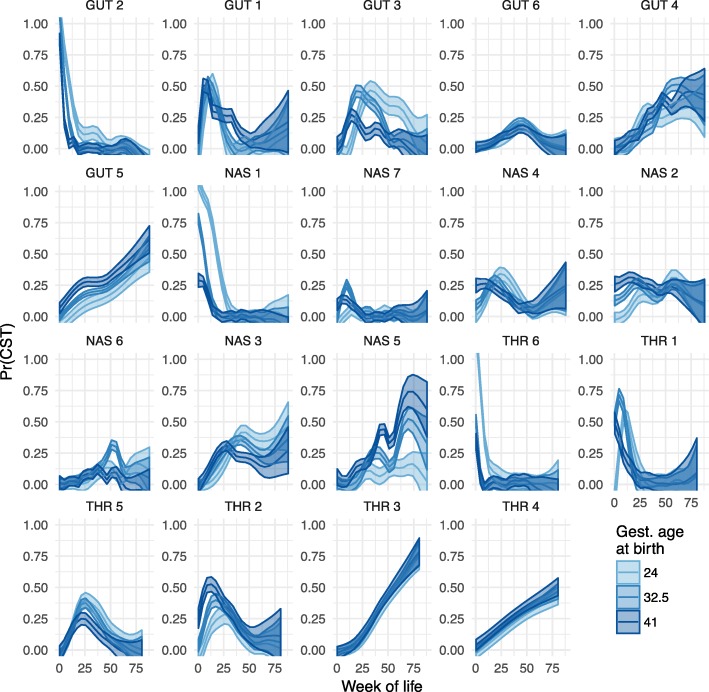
Associations between community state type membership and time. The posterior probability of membership to each CST (y-axis) is plotted over weeks of life (x-axis), estimated as a non-parametric function of week of life and gestational age at birth. The CSTs are sorted by post-menstrual age at which they achieve a maximal probability of occurrence

In order to elucidate the relationship between the temporal components of host maturity and the progression of community types at each body site, we further examined the associations between CSTs and maturity (GA) at birth and age, which can be measured developmentally as PMA or postnatally with WOL. We first fit smoothed curves of the probability of being in a given CST against WOL and GA at birth (Fig. 3) and the probability of being in a given CST against PMA and GA at birth (Additional file 7: Figure S6), stratifying subjects by GA at birth in both cases. This revealed three distinct canonical temporal patterns of CST occurrence (see the “Methods” section). The first pattern*, chronological*, observed in 6/19 of the CSTs identified, was characterized by occurrence at consistent post-natal intervals and frequencies which showed no substantive difference over the first year of life between pre- and full-term infants (e.g., throat CST 3 and gut CST 1), indicating that the week of life, a proxy for development shaped by environmental exposure, drives the occurrence of these community types. For these CSTs, the occurrence probability curves in Fig. 3 for each birth stratum overlap. By contrast, the second observed occurrence pattern was *idiosyncratic to maturity at birth*; whereby CSTs occurred at characteristic post-natal intervals, but their frequency of occurrence was dependent on gestational age at birth and they were significantly over- or under-represented in pre-term subjects (e.g., nasal CSTs 2 and 5). In Fig. 3, these CSTs’ curve for one maturity stratum reaches a distinctly higher peak than at least one other maturity stratum. Lastly, a *convergent* pattern of occurrence was observed in the 9/19 of CSTs. These CSTs showed increased probabilities of occurrence at earlier post-natal ages in full-term infants, but their occurrence probabilities in pre-term infants reached parity after a post-natal interval approximately equal to their prematurity (e.g., gut CST3, throat CST1, and nasal CST4). Infants with equal CST probability tend to have similar post-menstrual ages. The convergent pattern of occurrence implicates PMA, a proxy for host innate developmental maturity, as a driving force. The curves for the most and least mature strata for these CSTs in Fig. 3 have peaks of similar height that are offset along the week of life axis.

We then constructed a single index model of age for each CST, which fit the probability of observing the CST as a function of a pseudo time index that is learned by the model. This pseudo time is a weighted average of GA at birth and WOL (Additional file 8: Figure S7). The GA at birth and WOL weights that form the pseudo time index quantify the tradeoff between time spent pre- and post-natally, with respect to the probability of manifesting a given CST. These models confirmed the trends described above, with three basic patterns being observed (see the “Methods” section).

### Associations between community types and composition across body sites

**Fig. 4:**
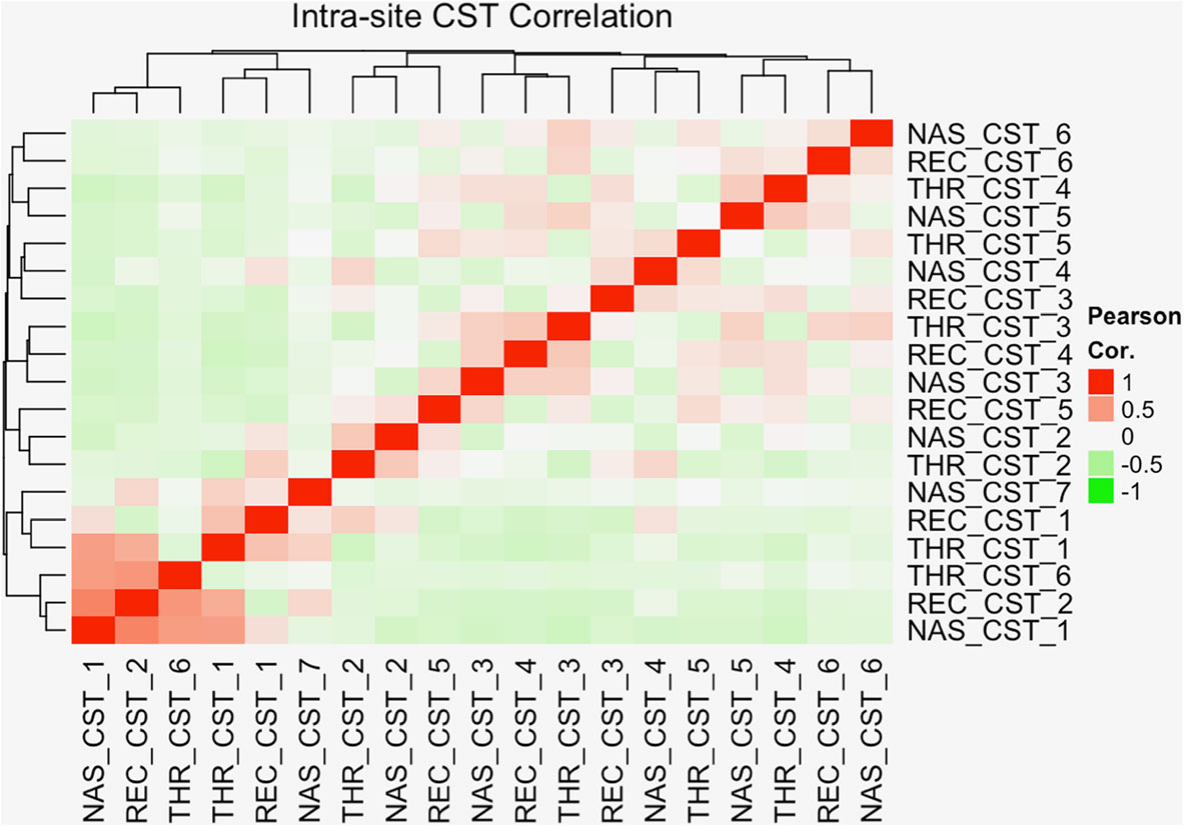
Pairwise correlations between community state types (CSTs) at different body sites. CSTs on the x- and y-axes are identified by body site nasal (NAS), throat (THR), gut (REC) and type. Each cell represents the Pearson sample correlation of CST membership probability across body sites in the same individual. Red-hued cells correspond to positive correlation coefficients. CST co-occurrence is non-independent with the CST of one body site highly predictive of the CSTs of the other two body sites

Having established a summary narrative of typical microbiota development within each body site and identified host age and developmental maturity as primary driving factors, we sought to explore the possibility of significant associations between microbiota across body sites. Given that time is a common factor driving development host-wide, our expectations were confirmed in that the co-occurrence patterns of CSTs across body sites were significantly non-independent, as assessed by a chi-squared test (*p* value < 0.001). To further characterize these associations, we calculated the pairwise correlations between CSTs observed at each site (Fig. 4). Again, co-occurrence patterns between sites were highly significant, suggesting that the observation of a given CST at one body site is predictive of the concurrent CSTs at other body sites. The greatest degree of CST correlation between body sites was observed among nasal CST 1, gut CST 2, and throat CSTs 1 and 6, for which all cross-body site pairs were positively correlated.

Given the strong associations at all body sites between CST occurrence and infant developmental and chronological age, correlations across body sites were expected. We sought to isolate these correlations resulting from host-wide temporal influences and assess remaining associations between community composition across sites. In order to control for time and other confounding factors and identify potential associations arising from direct or indirect interactions across sites, we further assessed the associations between the CST of each body site and the microbiota composition of the other body sites using a series of linear regression models. As predictors for the abundance of each taxon in a given body site, we first used mode of delivery, GA at birth, birth season, and day of life (which was modeled with a natural spline to allow for non-linear effects), as well as a per-subject random effect to account for repeated sampling of the same individuals. After fitting these “null” models, which contained no information about the composition of other body sites, we then added additional predictive terms for the CSTs of the other body sites and refit the “full” models. Because we sought to identify the relationships across body sites that could not be explained by infant maturity alone, we called significant only those associations between taxon abundance and remote CST for which inclusion of the remote CSTs as terms in the model significantly improved its explanatory power, after multiple test corrections (FDR ≤ 0.05, see the “Methods” section). We identified significant associations across all pairs of body sites (Additional file 9: Table S2), with the most significant associations identified between CSTs of the nose and gut and taxa in the throat. Fewer associations were significant between the gut and nose. Within each body site, certain taxa were uniquely associated with the CST of only one of the other body sites, while other taxa exhibited significant associations with the CST of both of the other two body sites. Of the additional variables included in the models, only those related to maturity were highly significant, with the day of life splines having the largest number of significant associations, followed by gestational age at birth. Mode of delivery had only four significant associations, all with taxa in the gut, while birth season had no significant associations (see Additional file 10: Figure S8 and Additional file 11: Table S3).

A number of taxa had significant associations with CSTs of other body sites at which the taxa themselves were not observed, ruling out direct exchange of these bacteria as the sole explanation for the associations. These taxa included *Bacteroides ovatus*, *Clostridium perfringens*, *Actinobaculum,* and *Faecalibacterium sp.* (Additional file 12: Table S4). Instead, these associations were consistent with the presence of bacteria in one site impacting, or being impacted by, development of microbiota at another site through indirect physiological or metabolic mechanisms. In order to assess cases where taxa were present in both associated sites, including *Viellonella*, *Prevotella*, and *Dorea*, we tested the OTU residuals for correlation after adjusting for PMA with a spline and each subject with a random effect. There were approximately fifty shared OTUs between each pair of sites, which on average were positively correlated for each site pair. The strongest correlation between shared OTUs was between the nose and throat, followed by the throat and gut, followed by the nose and gut (Additional file 13: Figure S9).

**Fig. 5:**
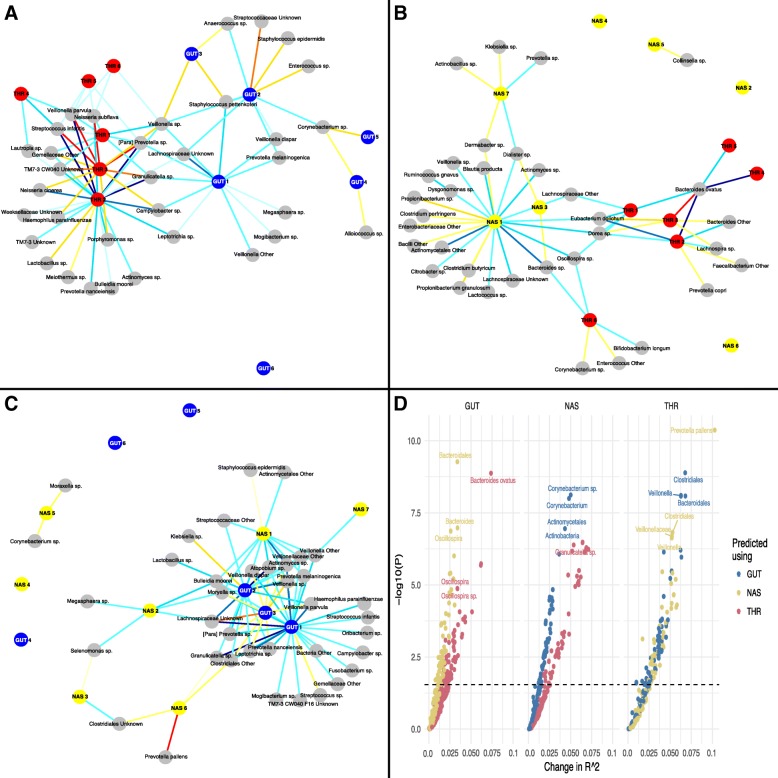
Significant associations between taxa abundance and community state type (CST) across body sites. A bipartite graph was used to visualize the associations between CSTs and taxa at a distal body site (nasal (**a**), gut (**b**),throat (**c**)), with significant associations at a false discovery rate (FDR) of 5%. Edges indicate significant associations with color marking the direction and significance of the effect (red: increase in abundance, blue: decrease in abundance). Color shade corresponds to the level of significance, with lighter colors being less significant. Nodes are positioned using a force-directed layout, which places taxa or CSTs with similar patterns of significant associations near each other while attempting to optimize readability and limit overlap. **d** Relationships between taxa abundance and CSTs was also visualized using a volcano plot, with improvement in explanatory power (*R*^2^) conferred by the inclusion of CSTs in the model on the x-axis and − log10 *p* values of the model improvement on the y-axis. With individual taxa in each body site as the outcome (subplots GUT, NAS, and THR), a linear regression model was fit using the with and without CSTs of the other body sites as covariates, controlling for gestational age at birth, day of life, mode of delivery, birth season, and subject-level random effects. Full models (including all CSTs) were tested against null models (excluding the CSTs of the other body sites in turn) with a series of *F* tests

The associations between each set of CSTs and specific taxa (Additional file 9: Table S2) was visualized in two ways. First, as a bipartite graph (Fig. 5a–c) in which each site’s most taxonomically specific significant taxa were connected to the CSTs of the distal body sites to which they had significant associations at a FDR of 5%. Edge color indicates the direction and significance of the association, either as a decrease or increase in abundance when the associated remote CST is observed. Second, as a volcano plot (Fig. 5d), this indicates the significance (*F* test *p* value) and the magnitude of the increase in explanatory power (change in *R*^2^) when the CSTs of distal body sites are added to the regression models that include as covariates mode of delivery, GA at birth, birth season, day of life (as a natural spline), and a per subject random effect, and taxon abundance as the outcome. We identified 105 taxa with significant cross-body site associations: 34 in the gut, 34 in the nose, and 37 in the throat, with some taxa being significant in multiple body sites where they occurred. In the gut, 5 taxa were significantly associated with CSTs in both the throat and nose, 12 taxa in the nose were associated with both gut and throat CSTs, and 23 taxa in the throat were associated with both nose and gut CSTs. Among taxa present in the gut, the largest numbers of associations were identified with nasal CST 1 and throat CST 2, with 22 and 7 taxa respectively, and the single most significant association was between *Bacteroides ovatus* and throat CST 2 (Fig. 5b, d). Notably, *B. ovatus* was not identified in throat samples but was present in 1% of nasal samples at a low (< 1%) abundance. Among taxa present in the nose, the largest numbers of associations were identified with gut CST 1 and throat CST 2, with 12 and 22 taxa respectively, and the single most significant association was between an OTU of *Prevotella* and throat CST 2 (Fig. 5a, d). Among taxa present in the throat, the largest numbers of associations were identified with nasal CST 1 and gut CST 1, with 12 and 26 taxa respectively, and the single most significant association was between *Prevotella pallens* and nasal CST 6 (Fig. 5c, d). In the gut, an OTU of *Dorea* exhibited the most significant associations, with six CSTs from the nose and throat found to be significant. In the nose, an OTU of *Veillonella* had the most associations, with seven CSTs from the gut and the throat. In the throat, the *Lachnospiraceae* family and an OTU of *Veillonella* had the most associations, each with six CSTs from the nose and gut.

### The microbiome is canonically correlated across body sites following time and space

These observations prompted us to assess the extent to which the OTU composition of each body site over time can be explained as a function of the OTU composition of the other body sites, without the dimension reduction associated with using CSTs. We again paired microbiome samples from different body sites that were acquired at the same visit for each participant, resulting in nasal-gut, nasal-throat, and rectal-throat site pairs. We then assessed the correlation of taxa between body sites directly using canonical correlation analysis (CCA) [31], which transforms two sets of multivariate observations into a series of *canonical correlates* (weighted averages) that maximize the score cross-correlations, quantifying the extent to which two sets of multivariate observations are correlated. Using a cross-validation scheme that held out blocks of individuals, we found in validation data that the subspace cross-correlations varied from 0.75 (nasal-throat) to 0.6 (gut-throat), for the first canonical coordinate (CC) (Additional file 14: Figure S10). Since we previously established that many OTUs vary as a function of time, we anticipated that temporal variation was responsible for much of this correlation. As expected, after adjusting for time by regressing out PMA in each site with a 14 degree-of-freedom spline, the time-stabilized correlations were attenuated in all site pairs, but still significantly different from zero in the nasal-gut and nasal-throat pairs (Additional file 14: Figure S10).

## Discussion

Development of the infant microbiome landscape is a critical factor in overall infant maturation and long-term health. In this study, we examined the progression of and spatiotemporal interactions between the gut and respiratory microbiota in pre- and full-term infants. We applied dimension reduction techniques and a longitudinal study design to reveal a summary narrative of infant microbiota development in terms of archetypal community states that have characteristic features of composition, with most CSTs being observed in most infants and occurring in a canonical pattern of temporal progression. By decomposing infant maturity into its component parts—post-menstrual age, post-natal age, and gestational age at birth—we show that different facets of the developmental phenotype are influenced in different ways by pre-term birth: some community states are delayed (convergent); some are over or under-represented—or even absent—depending on gestational age at birth (idiosyncratic to maturity at birth); and some are entirely unaffected by pre-maturity, with occurrence depending entirely on post-natal age (chronological). These findings suggest that the innate developmental programming of the human host and adaptive developmental processes shaped by environmental exposures act as distinct drivers of microbiota development with exclusive influences on certain aspects of the phenotype and combined influences on others. Elucidating these nuances of development and establishing a conceptually and analytically tractable representation of normal patterns of progression is essential for devising strategies of care and treatment that facilitate the establishment and maintenance of healthy respiratory and gastrointestinal tract microbiota in early life, especially in pre-term infants.

Knowledge of inter-site interactions between these anatomical niches forms the basis for understanding microbiota perturbations in sick infants and potential alterations in the microbiota of other sites. We apply an analytical framework using summary community state types combined with longitudinal modeling to identify significant associations between microbiota across the nose, throat, and gut during early life development in pre- and full-term infants. We demonstrate that the abundance of specific taxa in one body site exhibited strong associations with the community types of the other body sites. Using a natural spline function to control for time and linear regression to control for GA at birth, mode of delivery, birth season, and within-subject correlation, we found that incorporating the community types of the other body sites significantly improved the explanatory power of our model for the abundances of 105 unique taxa, many of which were present in only one member of a pair of associated body sites, ruling out the possibility of direct transmission as the mechanism of association. We then performed canonical correlation analysis to validate the explanatory power of the composition of each body site for every other body site. While the effect of time accounted for the majority of the canonical correlation between sites, a significant degree of correlation was observed even after controlling for temporal effects. These observations suggest a potential systemic coordination of microbial abundance and distribution across the infant microbiota landscape during early life development.

Early infant microbiota studies have focused on single anatomical sites, such as the gut and respiratory microbiota, with unique communities and distinct functions. Studies on pre- and full-term infant gut microbiota have shown that postnatal microbial colonization initiates maturation of infant intestinal structures and mediates the development of the immune system through interactions with gut epithelial, immune effector, and mucus-producing cells [32–34]. Deficiency in colonization of pre-term infant gut microbiota has been associated with delays in immune development, alterations in host metabolism, and inflammatory diseases such as necrotizing enterocolitis (NEC) [11, 35–39]. Longitudinal studies with pre-term infants have shown that the gut microbiota develops in a series of phases associated with postmenstrual age (PMA), more so than post-natal age, suggesting possible coordination between microbiota maturation and functional differentiation of the gut epithelium at defined stages of infant development [25]. Recent reports on neonatal respiratory microbiota have identified similar interactions of microbiota with mucosal epithelial and immune cells and an association with respiratory tract infections and chronic lung disease of prematurity [40–43]. However, most respiratory microbiome studies have focused on a limited number of samples from full-term infants. The influence of the respiratory microbiota on lung immunity and respiratory diseases in high-risk pre-term infants underscores the need to better understand initial microbial colonization and temporal dynamics of respiratory microbiota through longitudinal studies as we describe here.

The gut and respiratory tracts in infants share the same embryonic origin, with mucosal surfaces composed of columnar epithelial cells that sense the commensal microbiota and in turn shape local and systemic immunity as infants mature and as a function of PMA [32–34, 42, 44]. Changes in infant gut and respiratory microbiota that occur as a result of diet, antibiotics, therapeutics, and environmental exposures in the NICU are likely to influence the microbiota at both sites [25, 45, 46]. The effect of these changes can be illustrated by antibiotic-induced alterations of neonatal gut microbiota during the crucial early postnatal period of immune competence, which increase the risk of developing allergic airway disease and other atopies in subsequent childhood [47–49]. In adults, common chronic lung diseases, such as asthma and chronic obstructive pulmonary disease (COPD), often coincide with inflammatory bowel disease (IBD) and other chronic gastrointestinal syndromes [44, 50, 51]. The occurrence of these chronic lung diseases is accompanied by functional and structural changes in the intestinal mucosa and increased intestinal permeability, suggesting that interactions between these two distal sites through the gut-respiratory axis impact adult health and disease [50, 52]. These gut-respiratory interactions likely function on several levels, ranging from direct transfer of bacteria between these sites through reflux and aspiration to indirect effects from bacterial metabolic products or mucosal immune responses common to both the gut and respiratory tract [40, 42, 44, 53]. Taken together, these observations of common developmental origins for the gut and respiratory tracts, as well as inflammatory diseases that affect both sites, support potential systemic mechanisms that coordinate microbiota development at these distal sites in infants.

The microbiota samples for our study were collected as gut, nasal, and throat swabs from pre- and full-term infants. In a previous study, we established the taxonomic similarity of infant gut microbiota samples collected either as rectal swabs or from fecal material on a diaper [25]. When evaluating respiratory samples for this study and their relatedness to lung microbiota, we first considered potential acquisition routes for respiratory microbiota. The lung microbiota in healthy individuals is acquired by direct mucosal dispersion and micro-aspiration from the upper respiratory tract (URT) [54, 55]. The microbiota in these sites are taxonomically similar, albeit with differences within the URT subcompartments (nasal cavity, nasopharynx, oropharynx, and trachea) and lungs, a result of cellular and physiological features, such as oxygen and carbon dioxide tension, pH, humidity, and temperature that distinguish these environments and select for particular taxa [54–56]. The nasopharynx and oropharynx are the primary sources of lung microbiota in infants, likely due to the anatomy of the infant URT and increased production of nasal secretions, both of which enhance dispersal of microbiota to the lungs [57, 58]. With the infant nasopharynx and oropharynx, a primary source of colonizing infant lung microbiota, the nasal and throat samples used in our study as representative proxies of the neonate lung microbiota identified significant associations of taxa and CSTs within the gut-respiratory axis. The substantial degree of statistically independent variation of the nasal and throat microbiota is noteworthy, suggesting that additional analysis of both sites will provide unique insights into gut-respiratory interactions.

In our initial observations of the CST microbiota content relative to PMA, we noted that *Staphylococcus* was the most abundant taxon in the first CST of all three body sites (Fig. 1 and Additional file 1: Figure S1). Subsequent CSTs in all three sites demonstrated a rapid decrease in *Staphylococcus* abundance, which was progressively replaced by site-specific taxa with cellular and metabolic capabilities required for adaptation to the developing host site and interaction with the colonizing microbiota. Previous studies of infant gut microbiota identified *Staphylococcus* as an early microbiota colonizer, with abundance determined by nutrition and mode of delivery [8, 59]. With a metabolism biased towards carbohydrate metabolism, emerging data suggests the potential for a strong impact of *Staphylococcus* on disease programming and obesity in later life [60–62]. In vitro and in vivo animal experiments assessing transcriptomic and phenotypic responses of *S. aureus* to microbiota partners have revealed mechanisms that modulate metabolism, virulence, and survival in a multi-species bacterial community [63–65]. Similar experimental approaches to study interactions between members of the microbiota are needed to assess the mechanistic foundation of microbiota associations identified through computational means.

The temporal progression of CSTs and significant association between taxa and CST at multiple body sites suggested the potential for cross-site microbiota interactions during infant development. We identified one hundred five unique taxa with two forms of significant cross-body site associations: one with taxa present in the multiple body sites where the taxa-CST interaction occurred and a second with interacting taxa present only in one body site. Interactions between taxa present in both body sites were either proximally or distally related or both. For example, significant proximal associations were identified between *Veillonella parvulla* and *dispar* in the nasal-throat CST2 and throat-nasal CST1. Distal associations occurred between *V. dispar* nasal-gut CST2, and throat-gut CST1 and throat-gut CST2. *Veillonella* was identified as a core member of early infant microbiota in our analysis (Fig. 1, Additional file 4: Table S1, Additional file 1: Figure S1) and in multiple infant body sites in previous studies [6, 66]. The shared occurrence of *Veillonella* in proximal nasal and throat sites may be linked to early co-colonization of these environments by *Streptococcus*, which produces lactic acid, a carbon source for *Veillonella* [67]. Similar metabolic interactions that contribute to the temporal progression of CSTs and taxa-CST interactions will likely be identified through metabolic profiling of communities within each phase [68]. Significant cross-site associations with interacting taxa present at only one body site were demonstrated between *B. ovatus* in the gut and throat CST 2. These associations were consistent with the presence of bacteria in one site impacting, or being impacted by, the development of microbiota at another site through indirect physiological or metabolic mechanisms. Evidence that *B. ovatus*, a gut symbiont, digests polysaccharides in the gut as a carbon source for other members of the *Bacteroides* genus, places it at the center of cooperative ecosystem that is likely a central factor for gut microbiota functions and potential interactions with microbiota at distal sites [69–72]. Production of small chain fatty acids (SCFA) produced by *Bacteroides* and other enteric bacteria have been shown to profoundly affect both mucosal and systemic antibody responses [73]. Furthermore, the increased abundance of *B. ovatus* in the gut has been associated with systemic autoimmune diseases and IBD, a disorder linked to respiratory diseases as described above [74]. Overall, the identified taxa-CST associations have the potential to effect gut-respiratory crosstalk through the production of bacterial metabolites and ligands. In turn, dysbiosis of the gut microbiota can be anticipated to affect the dynamics of respiratory microbiota as well as systemic metabolic and immune responses [44].

Following observations made by other groups, we find that the strength of associations between microbial habitats is proportional to their proximity within the host [16]. The sites that are nearest to one another, such as the nose and throat, have the highest time-stabilized correlation and are more likely to share species in the canonical coordinate (CCA) site loadings. Distal pairs, such as the nose and gut have lower canonical correlations, with greater heterogeneity in the CCA loadings. In other words, body sites that are closer together have more microbial taxa in common and exhibit stronger associations between their microbiota composition than sites that are farther apart. These findings demonstrate that significant canonical correlations exist between the composition of microbial communities across body sites which cannot be entirely attributed to each body site’s independent temporal progression or to the repeated sampling of the same individuals.

Previous studies have demonstrated the influence of several external factors on infant microbiota development, including parental contact, mode of delivery, date or season of birth, nutrition, and exposure to the NICU environment [12, 25, 44, 58–60]. In our study, we included mode of delivery and season of birth in our analysis, neither of which had a significant association with CST progression (Additional file 10: Figure S8). However, we did not collect microbiota samples from parents or the NICU to assess the potential contribution of the microbiota background to CST progression, which is a limitation of our study. Because we are unable to sample from full-term infants in utero at PMA < 37 weeks, another potential limitation is the inability to directly compare pre- and full-term infants within the PMA 24–36-week window when pre-term infants are exposed to the NICU environment. In our approach, we avoid the issue of missing comparisons that arises from using post-menstrual age as the singular metric of maturity by comparing pre- and full-term samples using a multivariate representation of age comprised of gestational age at birth and day of life. Our analyses of CST occurrence patterns isolates the influence of both aspects of host maturity and distinguished between their exclusive and joint effects.

## Conclusion

Understanding the variation between and within subjects, conditions, and over time as the manifestation of compositionally distinct archetypal community state types provides an attractive conceptual and analytical framework for studying the microbiome [75]. While the extent to which community types are discrete or simply represent dense locations on a continuous gradient appears to vary depending on the conditions being sampled and the definition of “community type,” both the theoretical basis for community types and the utility of a community type-based framework have been established [75–83]. CST classification provides a conceptually and analytically tractable representation of the state of the microbial community within an individual at a particular body site and a particular time point, which captures information about the approximate abundance of distinguishing taxa as well as the presence of characteristic motifs of community makeup. The longitudinal series of observed CSTs provides a readily interpretable and comprehensible summary of the narrative of microbiota development, preserving information about the temporal representation of prominent taxa, and serving as a proxy for microbiota maturity and maturation rate. Such a framework offers a workable and intelligible representation of the archetypal features and the course grained phenotype of the high dimensional microbial community and its temporal dynamics, which facilitates high-level characterization, analysis, and understanding of this complex and intricate system for which a comprehensive, precise, and nuanced description would be intractable and unwieldy. Furthermore, the utility of community types can be extended to the identification of associations across body sites. Ding and Schloss used Dirichlet-Multinomial mixture modeling to define canonical community types within 18 adult body sites independently and then demonstrated that while the community types across different body sites were dissimilar in composition, they were predictive of one another [76]. The occurrence of community types simultaneously in different body sites was highly non-independent, suggesting an unknown mechanism of coordination or interaction acting at a distance. However, the community type framework may mask important biological variability and lack of power to detect specific taxa that serve as superior phenotypic biomarkers [75]. The approaches taken in our work reported here largely mitigate these shortcomings, by employing a sampling scheme that was dense and evenly distributed over gestational ages at birth, weeks of life, and modes of delivery, thereby making it unlikely that apparent community clusters are the result of a failure to observe intermediate points along a gradient. Our subsampling procedure to determine the number of clusters yielded a robust and parsimonious description of the data. Community types were not confounded within individuals, but shifted in type over the period of observation for each individual and were seen across a plurality of individuals. In this setting, where we sought to characterize the development of respiratory and gastrointestinal microbiota over the first year of life, community types have provided a high-level description of the state and progression which facilitated the interrogation of associations of CSTs with developmental age (PMA), and post-natal chronological age (WOL) as well as cross body-site relationships.

In summary, new clinical strategies for establishing and maintaining a homeostatic microbiota are needed for neonates at risk for gut and respiratory dysfunction and immune deficiencies. A greater understanding of infant respiratory microbiota colonization, interactions between the respiratory and gut microbiota, and possible developmental coordination between the two body sites are crucial steps in that direction. Our results demonstrate the existence of a host-wide network of associations between microbiota. The fact that these associations cannot be entirely explained by time, subject, or direct exchange of bacteria suggest unobserved factors mediating microbial dynamics and associations between microbiota across environments and at substantial distances. To our knowledge, these observations directly implicate, for the first time, a body-wide systemic mechanism coordinating the abundance and distribution of microbiota during early life development. The methods employed here may facilitate future efforts to evaluate disease, developmental maturity, therapeutic interventions, and dynamic interactions between multiple microbial communities and host systems.

## Methods

### Clinical methods

All study procedures were approved by the University of Rochester Medical Center (URMC) Internal Review Board (IRB) (Protocol # RPRC00045470) and all subject’s caregivers provided informed consent. The infants included in the study were from the University of Rochester Respiratory Pathogens Research Center PRISM study and cared for in the URMC Golisano Children’s Hospital Gosnell Family Neonatal Intensive Care Unit (NICU) or in the normal newborn nurseries and birthing centers. We sampled 1079 gut (279 from NICU and 800 post-discharge), 1013 nasal (262 from NICU and 751 post-discharge), and 538 throat (172 from NICU and 366 post-discharge) microbiota samples longitudinally from 38 pre-term and 44 full-term infants. Fecal (rectal), nasal, and throat material was collected from pre-term infants from 23 to 37 weeks GA at birth (GAB) weekly until hospital discharge and then monthly through 1 year of age, adjusted for prematurity. Rectal, nasal, and throat samples were collected from full-term infants at enrollment and monthly through 1 year. Each rectal sample was collected by inserting a sterile, normal saline moistened, Copan® flocked nylon swab (Copan Diagnostics, Murrieta, CA) beyond the sphincter into the rectum and twirling prior to removal. Nasal and throat samples were similarly collected by inserting and twirling a sterile, moistened swab into the throat or anterior nostril. Each swab was then immediately placed into sterile buffered saline and stored at 4 °C for no more than 4 h. Samples were processed daily, with the extraction of the fecal, nasal, and throat material from the swabs in a sterile environment and immediate transfer − 80^o^ C storage until DNA extraction.

### Exclusion of samples in which evidence of acute respiratory illness was observed

Samples collected during monthly visits in which evidence of acute respiratory illness was observed by the parents were excluded from the analysis. Each subject’s parent or primary caregiver was given a symptom COAST (Childhood Origins of Asthma) [84] score sheet, instructed on the use of the score and to notify the study team if the infant had symptoms that reached a score of 3 or greater. In addition, at every routine visit symptom questions were asked and if a child had symptoms that reached a score of 3 or greater the visit was converted from a well visit to an illness visit.

### Genomic DNA extraction

Total genomic DNA was extracted from the nose, throat, and rectal samples using a modification of the Zymo Fecal/Soil Microbe Miniprep Kit (Zymo Research, Irvine, CA) and FastPrep mechanical lysis (MPBio, Solon, OH). 16S ribosomal DNA (rRNA) was amplified with Phusion High-Fidelity polymerase (Thermo Scientific, Waltham, MA) and dual-indexed primers specific to the V3-V4 (319F: 5′ ACTCCTACGGGAGGCAGCAG 3′; 806R: 3′ ACTCCTACGGGAGGCAGCAG 5′) and V1-V3 (8F: 5′ AGAGTTTGATCCTGGCTCAG 3′; 534R: 3′ ATTACCGCGGCTGCTGG 5′) hypervariable regions [85]. Amplicons were pooled and paired-end sequenced on an Illumina MiSeq (Illumina, San Diego, CA) in the University of Rochester Genomics Research Center. Each sequencing run included (1) positive controls consisting of a 1:5 mixture of *Staphylococcus aureus*, *Lactococcus lactis*, *Porphyromonas gingivalis*, *Streptococcus mutans*, and *Escherichia coli* genomic DNA and (2) negative controls consisting of sterile saline.

### Microbiome background control

The background microbiome was monitored at multiple stages of sample collection and processing. All sterile saline, buffers, reagents, plasticware, and flocked nylon swabs used for sample collection, extraction, and amplification of nucleic acid were UV irradiated to eliminate possible DNA background contamination. Elimination of potential background from the irradiated buffers, reagents, plasticware, and swabs was confirmed by 16S rRNA amplification. After sample collection, multiple aliquots of sterile saline with swabs used for sample collection were carried through our entire sequencing protocol as individual samples, including DNA extraction, 16S rRNA amplification, library construction, and sequencing to monitor potential background microbiome. Data from these background control samples is deposited in SRA along with positive controls.

### 16S rRNA sequence processing

Raw data from the Illumina MiSeq was first converted into FASTQ format 2 × 300 paired-end sequence files using the bcl2fastq program, version 1.8.4, provided by Illumina. Format conversion was performed without de-multiplexing, and the EAMMS algorithm was disabled. All other settings were default. Sequence processing and initial microbial composition analysis were performed with the Quantitative Insights into Microbial Ecology (QIIME) software package [86], version 1.9.1. Reads were multiplexed using a configuration described previously [85]. Briefly, for both reads in a pair, the first 12 bases were a barcode, which was followed by a primer, then a heterogeneity spacer, and then the target 16S rRNA sequence. Using a custom Python script, the barcodes from each read pair were removed, concatenated together, and stored in a separate file. Read pairs were assembled using fastq-join from the ea-utils package, requiring at least 40 bases of overlap for V3 V4 sequences and 20 bases of overlap for V1 V3 sequence, while allowing a maximum of 10% mismatched bases. Read pairs that could not be assembled were discarded. The concatenated barcode sequences were prepended to the corresponding assembled reads, and the resulting sequences were converted from FASTQ to FASTA and QUAL files for QIIME analysis. Barcodes, forward primer, spacer, and reverse primer sequences were removed during de-multiplexing. Reads containing more than four mismatches to the known primer sequences or more than three mismatches to all barcode sequences were excluded from subsequent processing and analysis. Assembled reads were truncated at the beginning of the first 30 base window with a mean Phred quality score of less than 20 or at the first ambiguous base, whichever came first. Resulting sequences shorter than 300 bases or containing a homopolymer longer than six bases were discarded. Operational taxonomic units (OTU) were picked using the reference-based USEARCH (version 5.2) [87] pipeline in QIIME, using the May 2013 release of the GreenGenes 99% OTU database as a closed reference [88, 89]. An indexed word length of 128 and otherwise default parameters were used with USEARCH. Chimera detection was performed de novo with UCHIME, using default parameters [87]. OTU clusters with less than four sequences were removed, and representative sequences used to make taxonomic assignments for each cluster were selected on the basis of abundance. The RDP Naïve Bayesian Classifier was used for taxonomic classification with the GreenGenes reference database, using a minimum confidence threshold of .85 and otherwise default parameters [90].

### CST inference with Dirichlet-Multinomial modeling (DMM)

The DMM model was fit using the R package DirichletMultinomial version 1.16.0, R version 3.3.3. Sample composition was represented using normalized counts for each of the most specific operational taxonomic units (OTUs) present in at least 5% of the samples from a given body site. Normalization was performed on a per sample basis by taking the relative abundance of each OTU (after removing OTUs present in less than 5% of samples) and multiplying by 5000. Resulting non-integer counts were rounded down. In the DMM model, the number of Dirichlet components is a tuning parameter. For each body site, we used tenfold random subsampling of 80% of the samples to assess uncertainty in model fit for one through ten components, with the model fit being assessed as the Laplace approximation of the negative-log model evidence. We selected the number of components at each body site corresponding to the lower bound on the standard error of the model fit. We then fit complete models for each body site using all samples and the number of components selected and used the resulting posterior probabilities to assign each sample to a community state type (CST) corresponding to a Dirichlet component. The CSTs observed in each subject and at each body site over time are represented in Fig. 2, which was plotted using the TraMineR package, version 2.0-7. Color changes occur midway between consecutive samples of differing CSTs. Observation time points were quantized for plotting purposes only, and this was done by rounding down to the nearest whole week of post-menstrual age.

### Putative functional profiling and analysis

Putative functional profiles of all samples were generated using PICRUSt (version 1.1.3) [27] with the May 2013 version of GreenGenes, using default options, including PICRUSt’s precalculated files. CST functional enrichment was assessed using a Galaxy implementation of LEfSe (http://huttenhower.sph.harvard.edu/galaxy/) [91]. Functional relative abundance was normalized on a per-sample basis to sum to one million. Each CST was assessed independently by grouping all samples from a given body site into members of that CST and not members of that CST. FDR values of 0.1 and an LDA threshold score of 2.0 were used.

### Generalized additive modeling of CST

Generalized additive models (GAMs) were fit using R package mgcv for each CST and site. The probability of being in a CST was modeled (on the linear probability scale) as a smooth function of week of life and GA at birth, and a random effect for each individual. Formally, for each CST, for individual *i* at time *t*, we fit:

1 $$ P\left({\mathrm{CST}}_{it}\right)\kern0.5em =\kern0.5em f\left({\mathrm{WOL}}_{it},\kern0.5em {\mathrm{gaBirth}}_{it}\right)\kern0.5em +\kern0.5em {\mathrm{participant}}_i\kern0.5em +\kern0.5em {\mathrm{error}}_{it}, $$

where *f*(WOL_*it*_, gaBirth_*it*_) is a smooth function of week of life and GA at birth, participant_*i*_ is a random intercept for each participant, and error_*it*_ represents independent, homoscedastic noise. We plotted the fitted CST probability, under model (1) over a range of weeks-of-life for several representative gestational ages, and then compared this estimate to a single index model.

The single index model restricts the smooth function *f* in model (1) to seek a common “time” variable that accounts for both time spent inside and outside the womb. Symbolically, we require

2 $$ f\left({\mathrm{WOL}}_{it},\kern0.5em {\mathrm{gaBirth}}_{it}\right)\kern0.5em =\kern0.5em f\left(a\cdot {\mathrm{WOL}}_{it}\kern0.5em +\kern0.5em b\cdot {\mathrm{gaBirth}}_{it}\right). $$

For instance, if a = 1 and b = 0, then GA has no effect on the CST trajectory, and when a = b, then time spent inside the womb has the same effect on the probability of belonging to a CST as time spent outside the womb.

The CST categories of chronological, convergent, and idiosyncratic were derived by binning the log2 ratio of b/a in Eq. (2). If |log_2_*b*/*a*| < 1, then we declare the CST to be convergent. If log_2_*b*/*a* <  − 1 and a logistic regression testing for association between any occurrence a subject and their GA at birth was not significant at *p* < 0.05, then we declare the CST to be chronological. Otherwise, we consider it to be idiosyncratic. Additional file 15: Table S5 lists the parameters used to categorize CSTs.

### CST and taxa regression

We paired microbiome samples from different body sites that were acquired at the same visit for each participant. This generated three pairs of sites. The nasal-rectal sites had the greatest number of matched sample pairs, with 82 participants having 951 pairs of samples, while the nasal-throat sites had the fewest, with 40 participants having 483 sample pairs. The rectal-throat sites had 491 sample pairs. We applied arcsine sqrt-transformation to stabilize the variance of relative abundance and then fit linear mixed effects models to the abundance using the CST of the other two sites as the primary variables of interest. We adjusted as potential confounders the mode of delivery, GA at birth and 14 degree-of-freedom spline for WOL. The subject ID served as a random intercept. Associations with the primary variables of interest were tested for all taxonomic levels and reported on the most specific taxon (or equally specific taxa) within a phylogenetic lineage. We report a Wald test for equality between the abundance in each CST and its grand mean abundance. Associations significant at 5% FDR, calculated per site, are shown as edges in Fig. 5, which itself was generated using R packages GGally version 1.3.2 and network version 1.13.0. An overall test for association between a site and a taxon was derived by conducting an *F* test of the model that dropped the CST of that site as predictors and the full model. The change in pseudo *R*^2^ reports the change in variance explained by the fixed effects in the null and full models [92].

### Canonical correlation analysis

The CCA function implemented in R base was used for canonical correlation analyses. We employed tenfold cross-validation. We fit the CCA on the CSS normalized [93], species-level OTU vector for each pair of sites on nine/tenths of the subjects, then calculated the subspace correlations on the 10% of withheld subjects. Two times the standard error of the mean of the held-out subspace correlation is shown in the shaded region of Additional file 14: Figure S10.

## Additional files

Additional file 1: **Figure S1.** Unweighted Unifrac principal coordinate analysis plots of all samples from each body site, colored by the community state type of the sample. (PDF 3130 kb)

Additional file 2: **Figure S2.** Dirichlet-Multinomial diagnostics including species level OTU relative abundance for each sample and CST. Panels A–C show the sqrt-transformed relative abundance for each sample, clustered into its CST group. OTUs were selected to have the largest between CST-variance. Sites have varying OTU depending if a taxa was present at a site. Thicker boxes to the right of the thin white lines show the Dirichlet-Multinomial parameter for the CST and OTU. Panel D shows the average abundance of selected genera across CST and body sites. Rows are *z*-scored. (PDF 719 kb)

Additional file 3: **Figure S3.** Goodness of fit for Dirichlet multinomial mixture models in 80% subsamples of data in gut (A), throat (B), and nasal (C). A series of models that included from 1 to 10 Dirichlet-Multinomial components were fit 10 times to datasets subsampled without replacement. The Laplace approximation to the Bayesian evidence was calculated for each subsample. (PDF 110 kb)

Additional file 4: **Table S1.** Genus level table indicating the average relative abundances of all taxa in each community state type. (XLSX 112 kb)

Additional file 5: **Figure S4.** Distribution of samples into community state types by PMA for (A) throat, (B) nasal, and (C) gut for pre-term and full-term infants. (PDF 130 kb)

Additional file 6: **Figure S5.** Effect sizes of significantly enriched metagenome pathways. Putative functional profiles of all samples were generated using PICRUSt. KEGG pathways that were enriched across CST were ordered by *p* value across all sites and CST. The top eight pathways for each site and CST were tabulated, which all had FDR-adjusted *p* values below 10%. The LDA effect size, providing a measure of discriminatory power, was used to perform hierarchical clustering of tabulated pathways across all sites’ CST simultaneously. (PDF 305 kb)

Additional file 7: **Figure S6.** Associations between CST membership and post-menstrual age. The posterior probability of membership to each CST (y-axis) is plotted over PMA (x-axis), as estimated as a non-parametric function of PMA and gestational age at birth. The CST are sorted by the post-menstrual age at which they achieve maximal probability of occurrence. (PDF 349 kb)

Additional file 8: **Figure S7.** Visualizations of models relating CST occurrence probability to post-natal and gestational age. (A) A bivariate probability model (model 1; see Methods) of observing each CST was fit for all gestational ages at birth between 24 and 43 weeks and the first 88 weeks of life. Each panel indicates the residual probability of observing a specific CST as a function of gestational age at birth (y-axis) and week of life (x-axis). Probabilities are represented as colored topographic maps, where whiter hues indicate higher probability of observing a CST relative to the overall mean. Contour lines are periodically labeled to provide the precise residual probabilities. Black dots indicate sample collection points, with time points beyond the final observations left blank (upper right of each plot). The black, dashed, diagonal lines indicate post-natal intervals at which CST occurrence probabilities are equivalent for all gestational ages at birth according to a single index model (model 2) relating maturity to CST occurrence probability. The single index model fits the probability of CST occurrence as a function of a linear combination of gestational age at birth and week of life, yielding a “single index” of maturity that accounts for time spent in and outside the womb. Concordance between the diagonal dashed lines and the topographical contour lines corresponds to the goodness of fit of the single index model. (B) Each CST (arrayed along the y-axis) has a distinct single index of maturity, which combines time spent in and outside the womb in a ratio that best explains the observed patterns of occurrence. This ratio of post-natal age (week of life) to gestational age at birth is plotted along the x-axis. A ratio of one (vertical dashed line) would indicate that time spent in utero is equivalent to time since birth (i.e., CST occurrence depends only on post menstrual age). (PDF 1247 kb)

Additional file 9: **Table S2.** Significant associations between taxon abundance at one body site and the CSTs of other body sites. NA’s represent instances where the full model including terms for CSTs from other body sites did not significantly improve the explanatory power of the null model, which did not include CST terms, at an FDR threashold of 0.05. Only associations in which at least one CST term had a *p* value of less than or equal to 0.05 are included. The values in the CST columns are the -log10(*p* value). Only associations at the most taxonomically specific level found are reported. (XLSX 29 kb)

Additional file 10: **Figure 8.** Coefficients versus *p* values for models that test OTU for associations with subject-level covariates. The taxa OTU regressions (see Methods “CST and Taxa regression”) included baseline models that contained the covariates *mode of delivery* (c-section vs vaginal delivery), *gestational age at birth* (gaBirth), and *birth season* as covariates. Coefficient estimates are shown versus the –log10 false discovery rate adjusted *p* values, adjusted jointly across all sites, coefficients and taxonomic levels. (PDF 148 kb)

Additional file 11: **Table S3.** Taxa associated with other covariates. (XLSX 477 kb)

Additional file 12: **Table S4.** Operational taxonomic units (OTUs) exhibiting a significant association with the CST of a body site in which they are not observed. (XLSX 10 kb)

Additional file 13: **Figure S9.** Distribution of correlations of OTU shared in common between body sites. PMA and a subject-level intercept are regressed out before calculating the Pearson sample correlation of each matched OTU. (PDF 67 kb)

Additional file 14: **Figure S10.** Canonical correlations between body sites. 10-fold crossvalidation allowed unbiased evaluation of the correlation (y-axis) in the first ten subspaces (xaxis) in held-out data (red) and the training data (blue). The correlation is attenuated after adjusting for PMA with a 14 and 25 degree-of-freedom natural spline. The 2 times the standard error of the mean of the cross-validation shows the sampling variability of the correlations. (PDF 43 kb)

Additional file 15: **Table S5.** Parameters for classifying CSTs into one of the three occurrence patterns. (XLSX 11 kb)

## Abbreviations

CST: Community state type
FDR: False discovery rate
GA: Gestational age
NICU: Neonatal intensive care unit
OTU: Operational taxonomic units
PMA: Premenstrual age
WOL: Week of life
