# Supplementary material for: Neonatal gut and respiratory microbiota: coordinated development through time and space
Source: Microbiome. 2018 Oct 26;6:193. doi: 10.1186/s40168-018-0566-5 (PMC6204011; doi:10.1186/s40168-018-0566-5)
Supplement: Supplementary file 1 — Figure S1. Unweighted Unifrac principal coordinate analysis plots of all samples from each body site, colored by the community state type of the sample. (PDF 3130 kb) [file 40168_2018_566_MOESM1_ESM.pdf]

Supplemental Figure 1

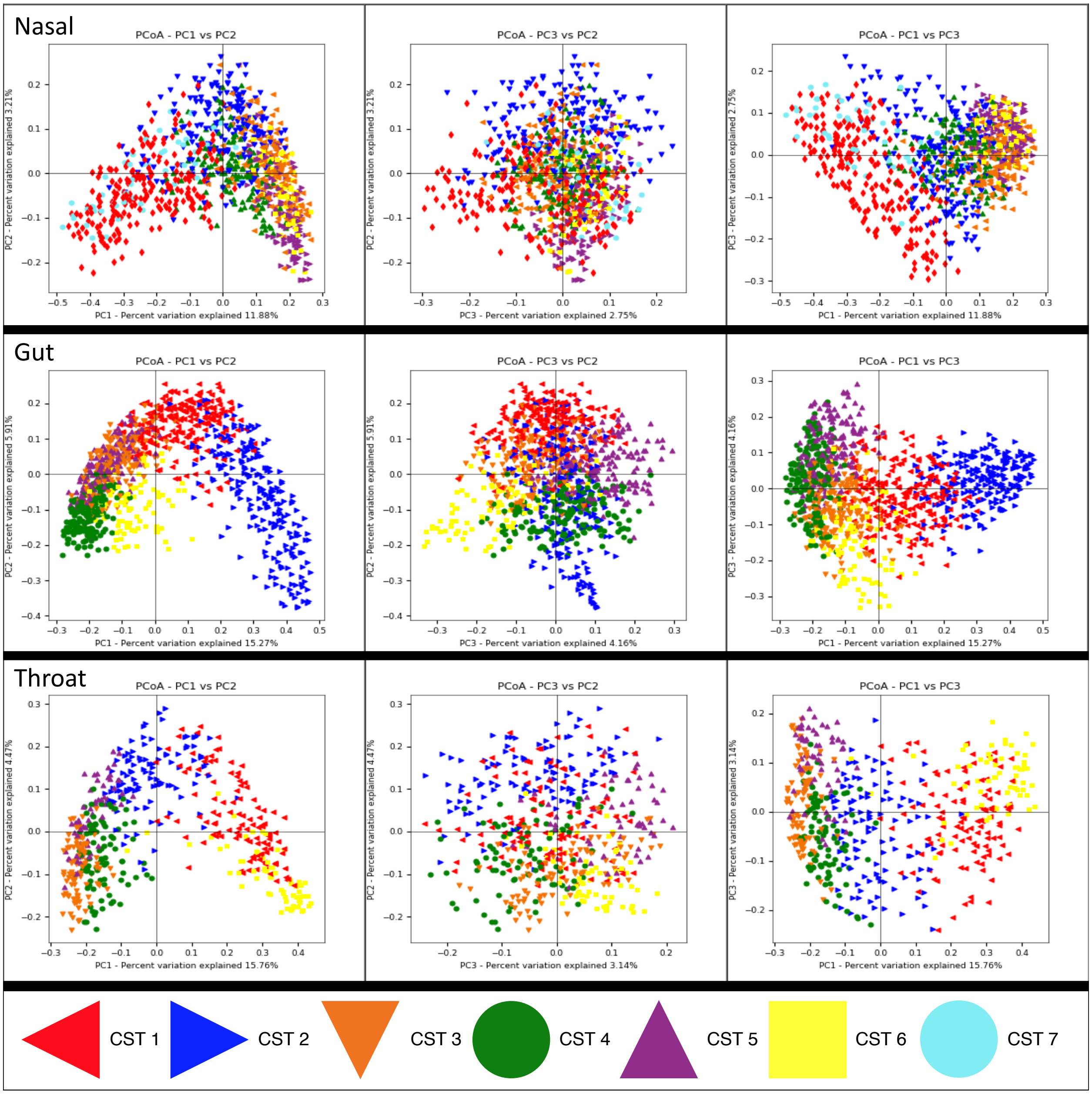

Supplemental Figure 1. Unweighted Unifrac principal coordinate analysis plots of all samples from each body site, colored by the community state type of the sample.
