# Supplementary material for: Neonatal gut and respiratory microbiota: coordinated development through time and space
Source: Microbiome. 2018 Oct 26;6:193. doi: 10.1186/s40168-018-0566-5 (PMC6204011; doi:10.1186/s40168-018-0566-5)
Supplement: Supplementary file 2 — Figure S2. Dirichlet-Multinomial diagnostics including species level OTU relative abundance for each sample and CST. Panels A–C show the sqrt-transformed relative abundance for each sample, clustered into its CST group. OTUs were selected to have the largest between CST-variance. Sites have varying OTU depending if a taxa was present at a site. Thicker boxes to the right of the thin white lines show the Dirichlet-Multinomial parameter for the CST and OTU. Panel D shows the average abundance of selected genera across CST and body sites. Rows are z-scored. (PDF 719 kb) [file 40168_2018_566_MOESM2_ESM.pdf]

Supplemental Figure 2

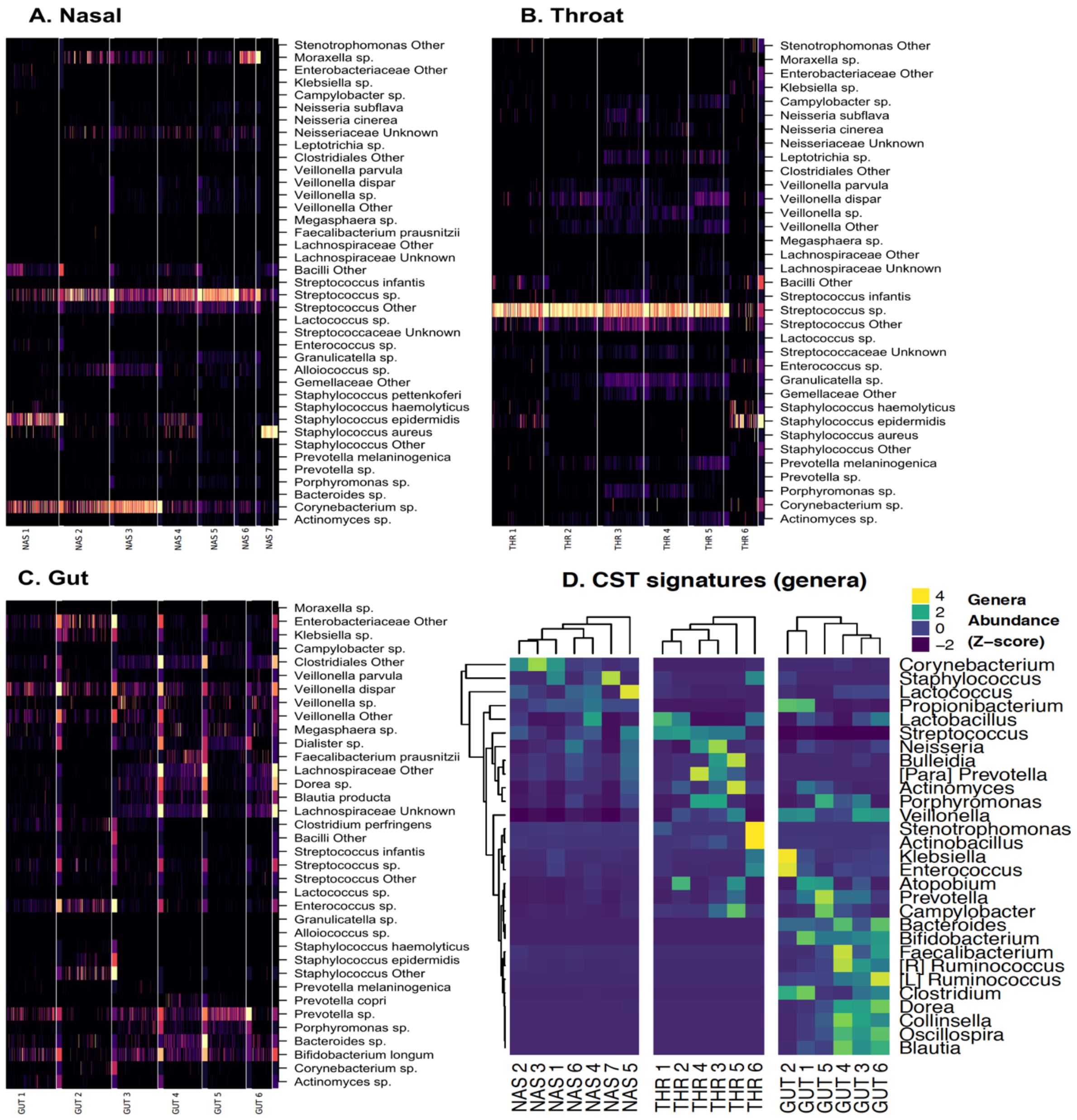

Supplemental Figure 2. Dirichlet Multinomial diagnostics including species level OTU relative abundance for each sample and CST.

Panels A-C show the sqrt-transformed relative abundance for each sample, clustered into its CST group. OTUs were selected to have the largest between CST-variance. Sites have varying OTU depending if a taxa was present at a site. Thicker boxes to the right of the thin white lines show the Dirichlet Multinomial parameter for the CST and OTU. Panel D shows the average abundance of selected genera across CST and body sites. Rows are z-scored.
