# Supplementary material for: Neonatal gut and respiratory microbiota: coordinated development through time and space
Source: Microbiome. 2018 Oct 26;6:193. doi: 10.1186/s40168-018-0566-5 (PMC6204011; doi:10.1186/s40168-018-0566-5)
Supplement: Supplementary file 3 — Figure S3. Goodness of fit for Dirichlet multinomial mixture models in 80% subsamples of data in gut (A), throat (B), and nasal (C). A series of models that included from 1 to 10 Dirichlet-Multinomial components were fit 10 times to datasets subsampled without replacement. The Laplace approximation to the Bayesian evidence was calculated for each subsample. (PDF 110 kb) [file 40168_2018_566_MOESM3_ESM.pdf]

Supplemental Figure 3

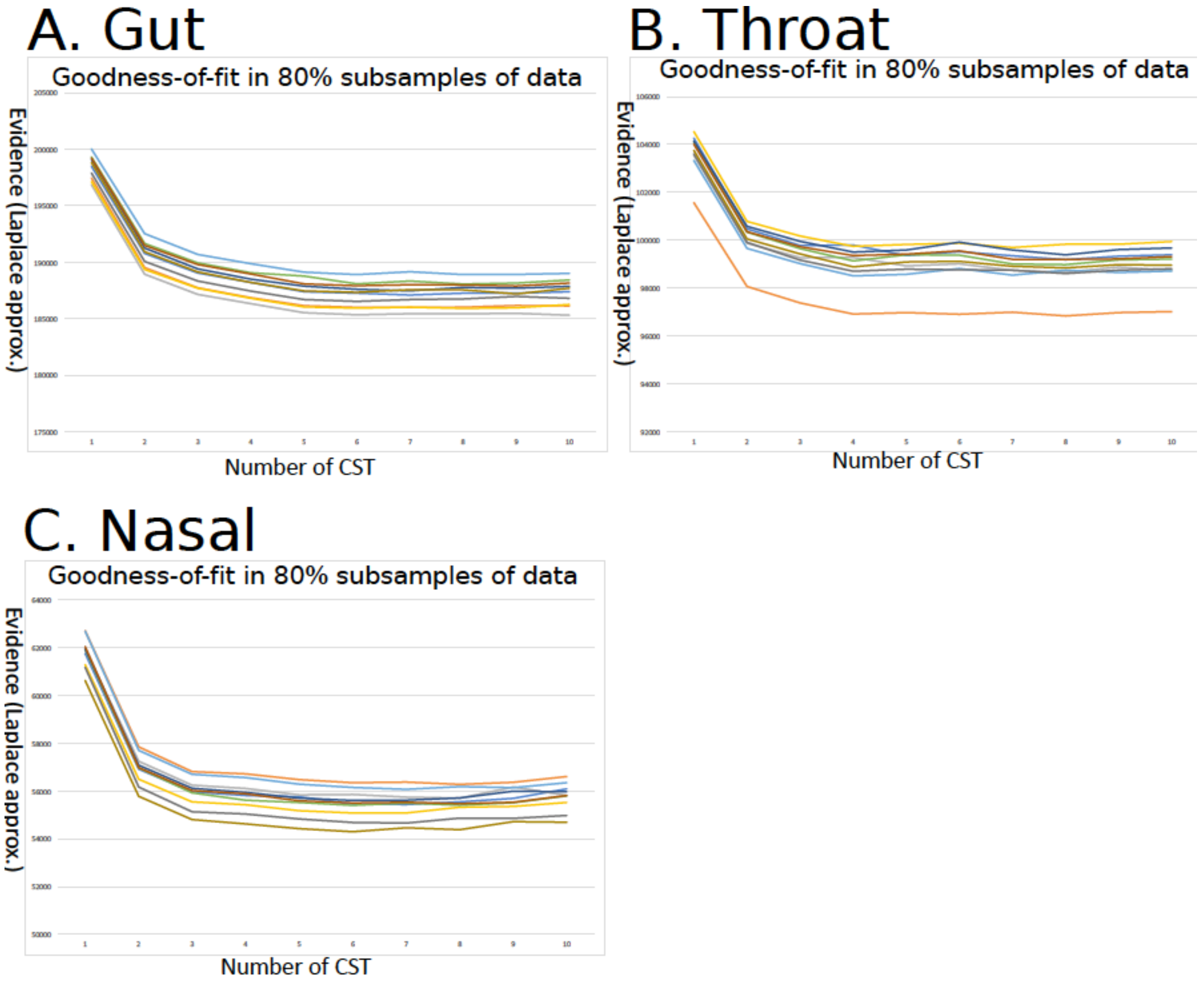

Supplemental Figure 3. Goodness of fit for Dirichlet multinomial mixture models in 80% subsamples of data in gut (A), throat (B) and nasal (C). A series of models that included from 1 to 10 Dirichlet-multinomial components were fit 10 times to datasets subsampled without replacement. The Laplace approximation to the Bayesian evidence was calculated for each subsample.
