# Supplementary material for: Neonatal gut and respiratory microbiota: coordinated development through time and space
Source: Microbiome. 2018 Oct 26;6:193. doi: 10.1186/s40168-018-0566-5 (PMC6204011; doi:10.1186/s40168-018-0566-5)
Supplement: Supplementary file 5 — Figure S4. Distribution of samples into community state types by PMA for (A) throat, (B) nasal, and (C) gut for pre-term and full-term infants. (PDF 130 kb) [file 40168_2018_566_MOESM5_ESM.pdf]

Supplemental Figure 4

A

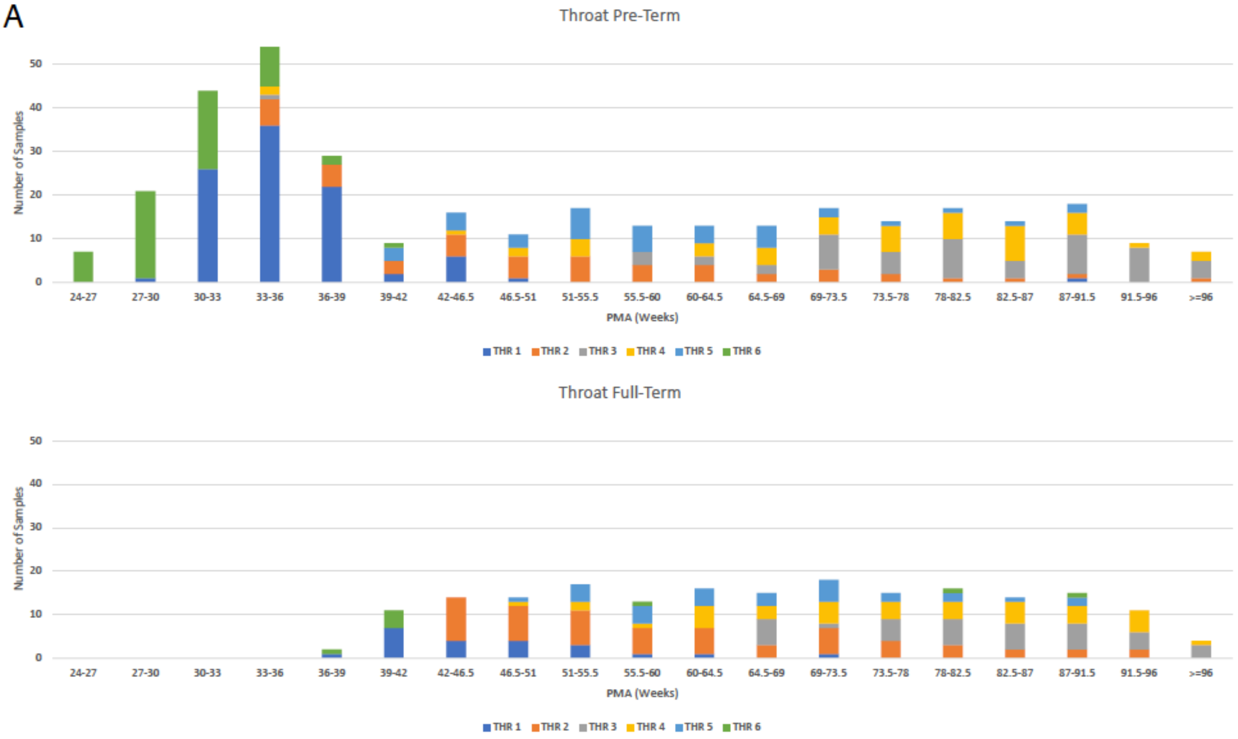

B

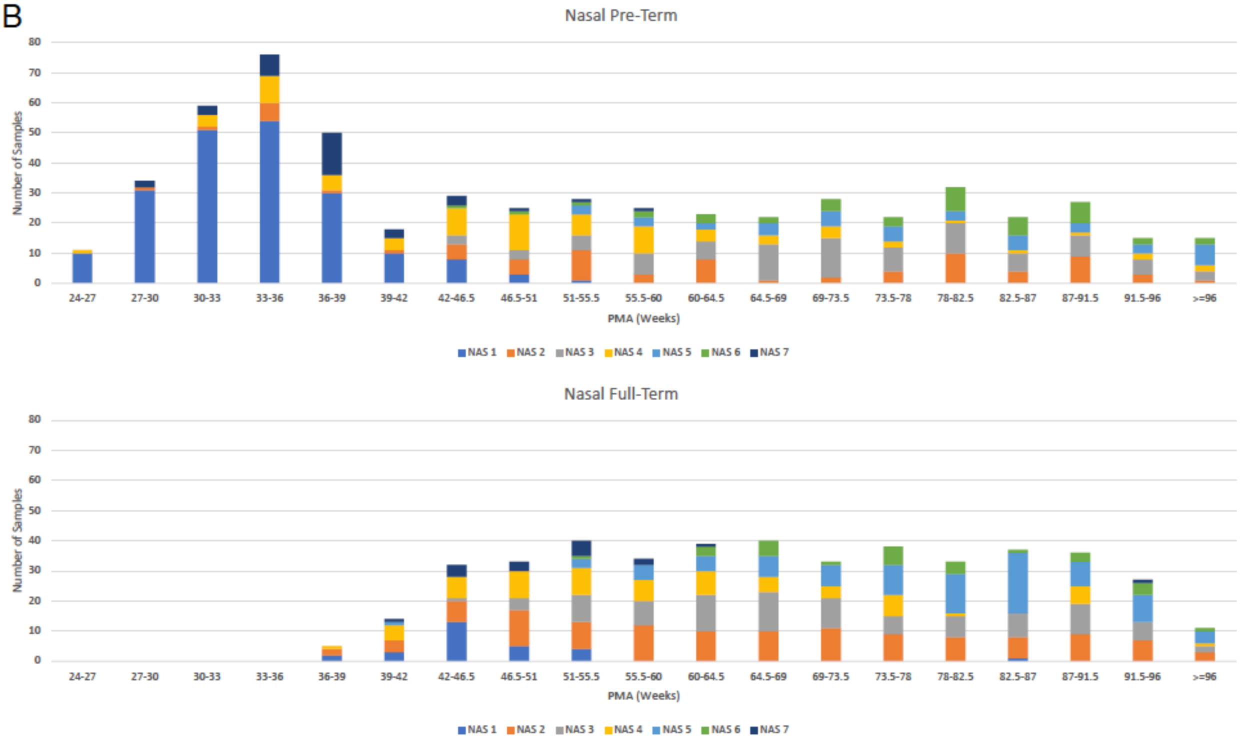

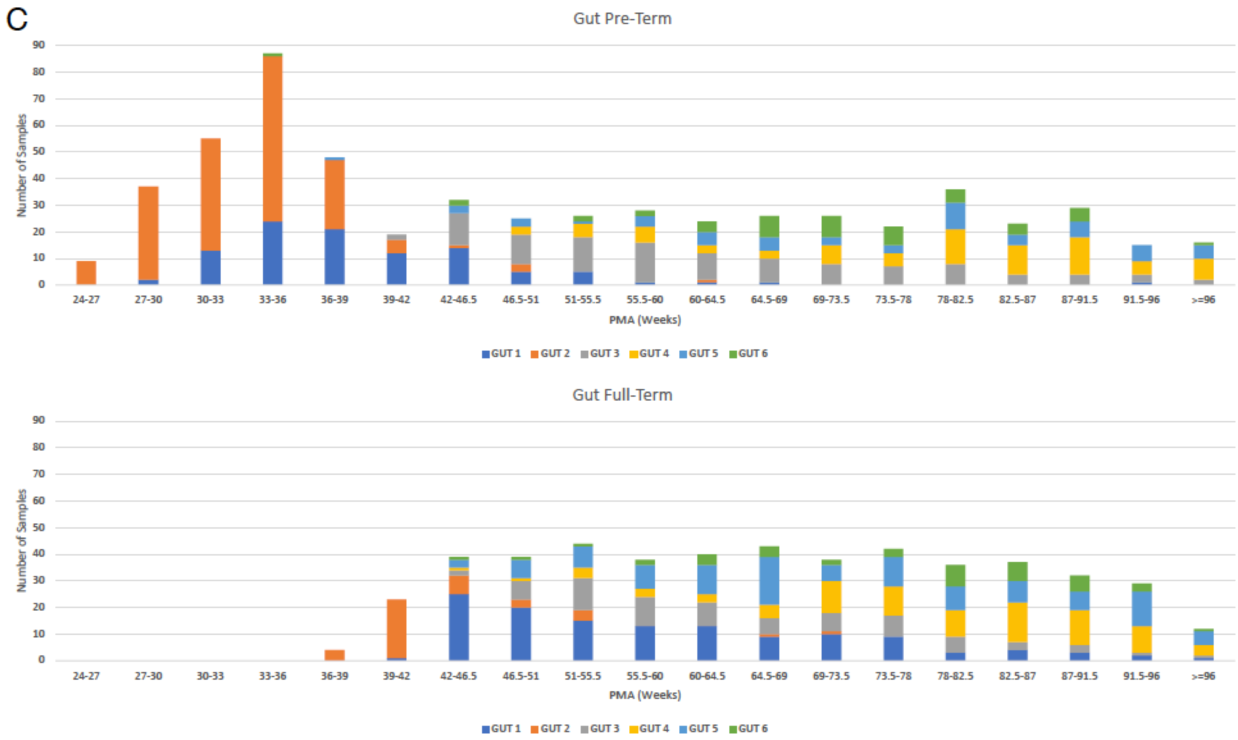

Supplemental Figure 4. Distribution of samples into community state types by PMA for (A) throat, (B) nasal and (C) gut for pre-term and full-term infants.
