# Supplementary material for: Neonatal gut and respiratory microbiota: coordinated development through time and space
Source: Microbiome. 2018 Oct 26;6:193. doi: 10.1186/s40168-018-0566-5 (PMC6204011; doi:10.1186/s40168-018-0566-5)
Supplement: Supplementary file 6 — Figure S5. Effect sizes of significantly enriched metagenome pathways. Putative functional profiles of all samples were generated using PICRUSt. KEGG pathways that were enriched across CST were ordered by p value across all sites and CST. The top eight pathways for each site and CST were tabulated, which all had FDR-adjusted p values below 10%. The LDA effect size, providing a measure of discriminatory power, was used to perform hierarchical clustering of tabulated pathways across all sites’ CST simultaneously. (PDF 305 kb) [file 40168_2018_566_MOESM6_ESM.pdf]

Supplemental Figure 5

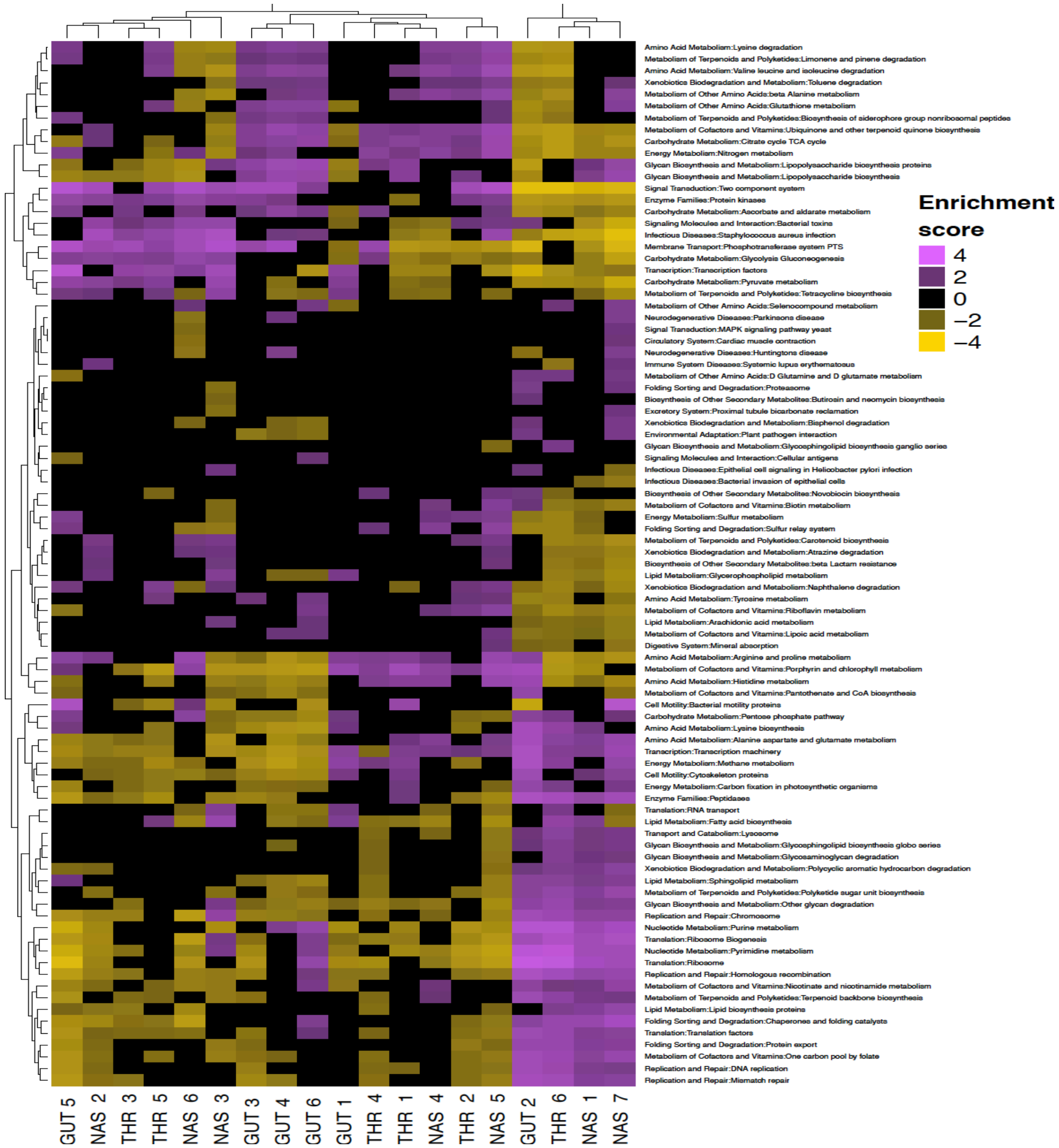

Supplemental Figure 5. Effect sizes of significantly enriched metagenome pathways. Putative functional profiles of all samples were generated using PICRUSt. KEGG pathways that were enriched across CST were ordered by p-value across all sites and CST. The top 8 pathways for each site and CST were tabulated, which all had FDR-adjusted p-values below 10%. The LDA effect size, providing a measure of discriminatory power, was used to perform hierarchical clustering of tabulated pathways across all sites' CST simultaneously.
