# Supplementary material for: Neonatal gut and respiratory microbiota: coordinated development through time and space
Source: Microbiome. 2018 Oct 26;6:193. doi: 10.1186/s40168-018-0566-5 (PMC6204011; doi:10.1186/s40168-018-0566-5)
Supplement: Supplementary file 7 — Figure S6. Associations between CST membership and post-menstrual age. The posterior probability of membership to each CST (y-axis) is plotted over PMA (x-axis), as estimated as a non-parametric function of PMA and gestational age at birth. The CST are sorted by the post-menstrual age at which they achieve maximal probability of occurrence. (PDF 349 kb) [file 40168_2018_566_MOESM7_ESM.pdf]

Supplemental Figure 6

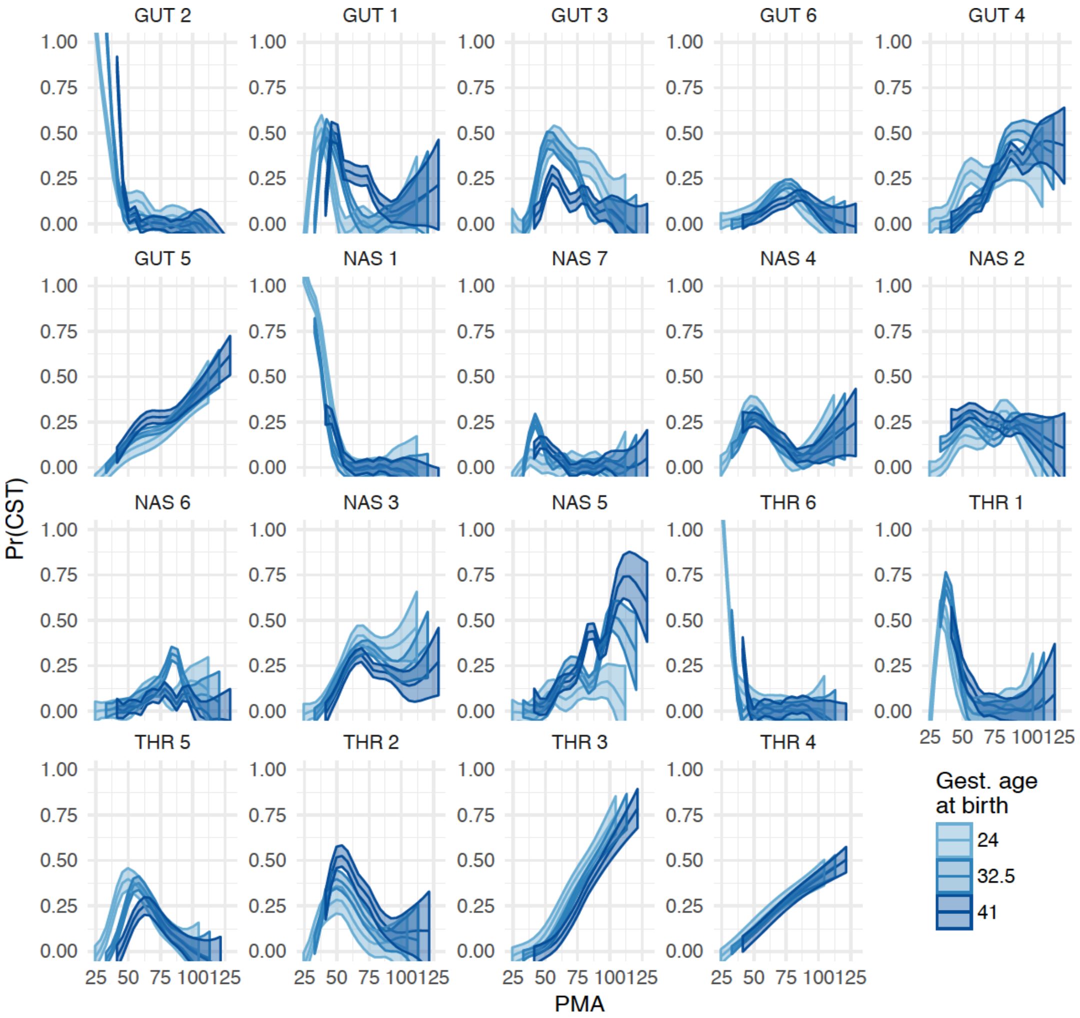

Supplemental Figure 6. Associations between CST membership and post-menstrual age. The posterior probability of membership to each CST (y-axis) is plotted over PMA (x-axis), as estimated as a non-parametric function of PMA and gestational age at birth. The CST are sorted by the post-menstrual age at which they achieve maximal probability of occurrence.
