# Supplementary material for: Neonatal gut and respiratory microbiota: coordinated development through time and space
Source: Microbiome. 2018 Oct 26;6:193. doi: 10.1186/s40168-018-0566-5 (PMC6204011; doi:10.1186/s40168-018-0566-5)
Supplement: Supplementary file 8 — Figure S7. Visualizations of models relating CST occurrence probability to post-natal and gestational age. (A) A bivariate probability model (model 1; see Methods) of observing each CST was fit for all gestational ages at birth between 24 and 43 weeks and the first 88 weeks of life. Each panel indicates the residual probability of observing a specific CST as a function of gestational age at birth (y-axis) and week of life (x-axis). Probabilities are represented as colored topographic maps, where whiter hues indicate higher probability of observing a CST relative to the overall mean. Contour lines are periodically labeled to provide the precise residual probabilities. Black dots indicate sample collection points, with time points beyond the final observations left blank (upper right of each plot). The black, dashed, diagonal lines indicate post-natal intervals at which CST occurrence probabilities are equivalent for all gestational ages at birth according to a single index model (model 2) relating maturity to CST occurrence probability. The single index model fits the probability of CST occurrence as a function of a linear combination of gestational age at birth and week of life, yielding a “single index” of maturity that accounts for time spent in and outside the womb. Concordance between the diagonal dashed lines and the topographical contour lines corresponds to the goodness of fit of the single index model. (B) Each CST (arrayed along the y-axis) has a distinct single index of maturity, which combines time spent in and outside the womb in a ratio that best explains the observed patterns of occurrence. This ratio of post-natal age (week of life) to gestational age at birth is plotted along the x-axis. A ratio of one (vertical dashed line) would indicate that time spent in utero is equivalent to time since birth (i.e., CST occurrence depends only on post menstrual age). (PDF 1247 kb) [file 40168_2018_566_MOESM8_ESM.pdf]

Supplemental Figure 7

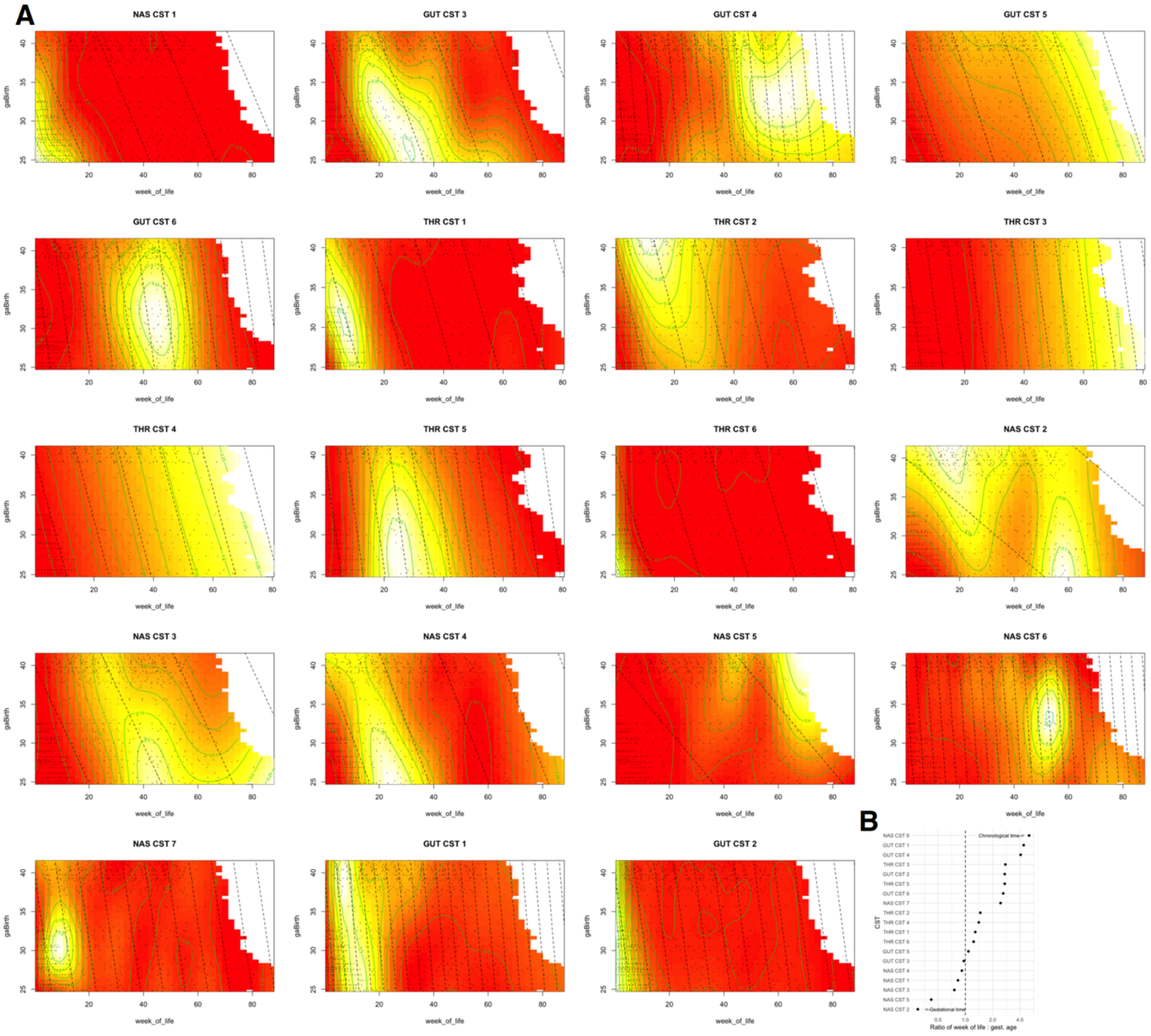

Supplemental Figure 7. Visualizations of Models Relating CST Occurrence Probability to Post-Natal and Gestational Age. (A) A bivariate probability model (model 1; see Methods) of observing each CST was fit for all gestational ages at birth between 24 and 43 weeks and the first 88 weeks of life. Each panel indicates the residual probability of observing a specific CST as a function of gestational age at birth (y-axis) and week of life (x-axis). Probabilities are represented as colored topographic maps, where whiter hues indicate higher probability of observing a CST relative to the overall mean. Contour lines are periodically labeled to provide the precise residual probabilities. Black dots indicate sample collection points, with time points beyond the final observations left blank (upper right of each plot). The black, dashed, diagonal lines indicate post-natal intervals at which CST occurrence probabilities are equivalent for all gestational ages at birth according to a single index model (model 2) relating maturity to CST occurrence probability. The single index model fits the probability of CST occurrence as a function of a linear combination of gestational age at birth and week of life, yielding a “single index” of maturity that accounts for time spent in and outside the womb. Concordance between the diagonal dashed lines and the topographical contour lines corresponds to the goodness of fit of the single index model. **(B)** Each CST (arrayed along the y-axis) has a distinct single index of

maturity, which combines time spent in and outside the womb in a ratio that best explains the observed patterns of occurrence. This ratio of post-natal age (week of life) to gestational age at birth is plotted along the x-axis. A ratio of one (vertical dashed line) would indicate that time spent in utero is equivalent to time since birth (i.e. CST occurrence depends only on post menstrual age).
