# Supplementary material for: Neonatal gut and respiratory microbiota: coordinated development through time and space
Source: Microbiome. 2018 Oct 26;6:193. doi: 10.1186/s40168-018-0566-5 (PMC6204011; doi:10.1186/s40168-018-0566-5)
Supplement: Supplementary file 10 — Figure 8. Coefficients versus p values for models that test OTU for associations with subject-level covariates. The taxa OTU regressions (see Methods “CST and Taxa regression”) included baseline models that contained the covariates mode of delivery (c-section vs vaginal delivery), gestational age at birth (gaBirth), and birth season as covariates. Coefficient estimates are shown versus the –log10 false discovery rate adjusted p values, adjusted jointly across all sites, coefficients and taxonomic levels. (PDF 148 kb) [file 40168_2018_566_MOESM10_ESM.pdf]

Supplemental Figure 8

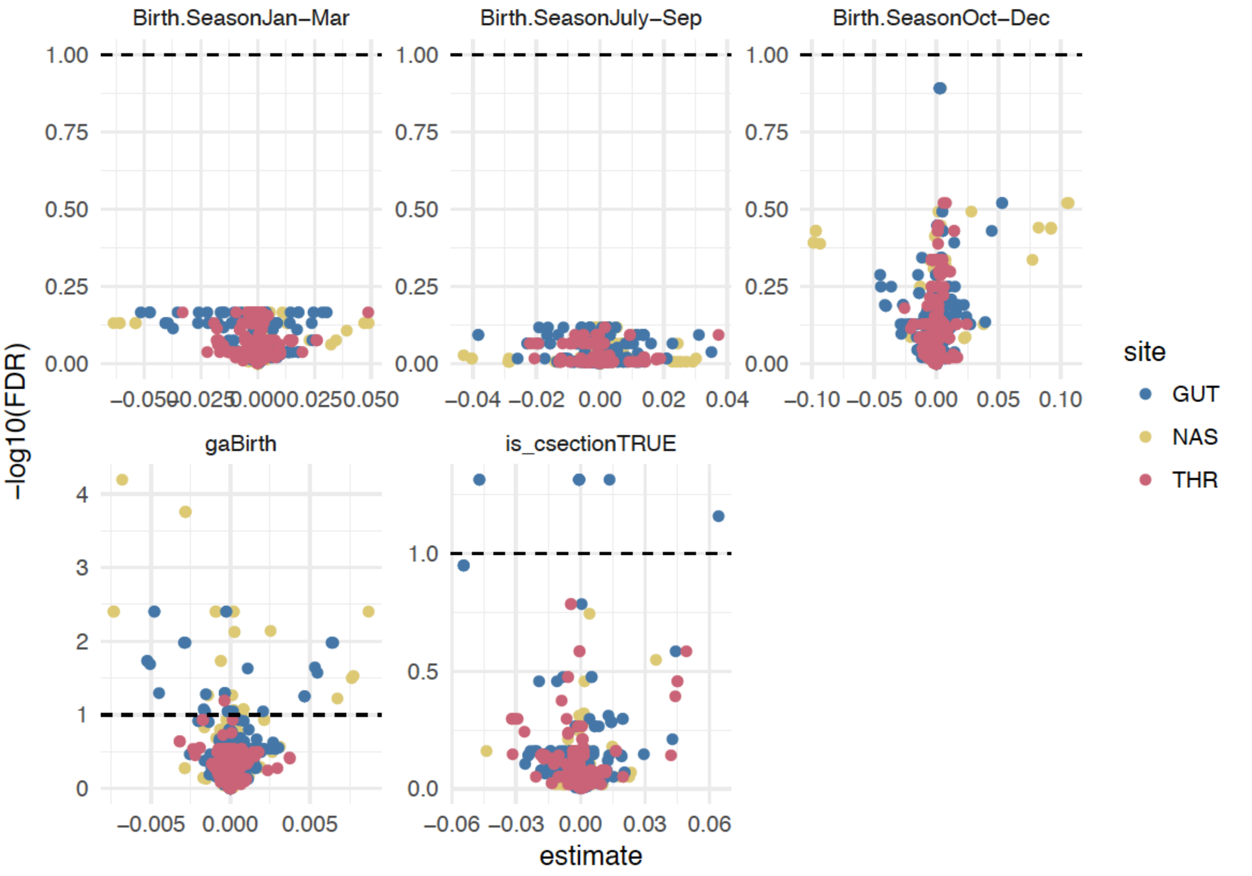

Supplemental Figure 8. Coefficients versus p-values for models that test OTU for associations with subject-level covariates. The taxa OTU regressions (see Methods “CST and Taxa regression”) included baseline models that contained the covariates *mode of delivery* (c-section vs vaginal delivery), *gestational age at birth* (gaBirth), and *birth season* as covariates. Coefficient estimates are shown versus the –log10 false discovery rate adjusted p-values, adjusted jointly across all sites, coefficients and taxonomic levels.
