# Supplementary material for: Neonatal gut and respiratory microbiota: coordinated development through time and space
Source: Microbiome. 2018 Oct 26;6:193. doi: 10.1186/s40168-018-0566-5 (PMC6204011; doi:10.1186/s40168-018-0566-5)
Supplement: Supplementary file 13 — Figure S9. Distribution of correlations of OTU shared in common between body sites. PMA and a subject-level intercept are regressed out before calculating the Pearson sample correlation of each matched OTU. (PDF 67 kb) [file 40168_2018_566_MOESM13_ESM.pdf]

Supplemental Figure 9

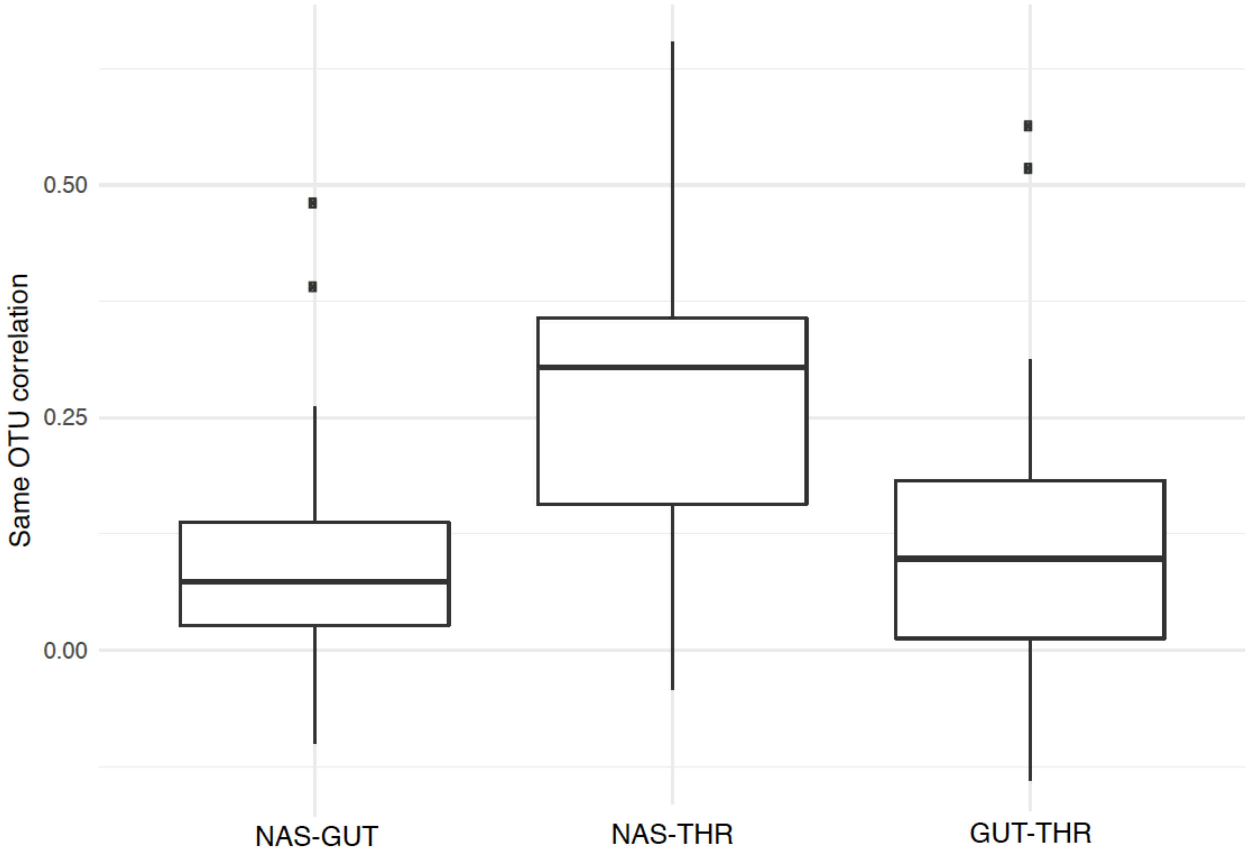

Supplemental Figure 9. Distribution of correlations of OTU shared in common between body sites. PMA and a subject-level intercept are regressed out before calculating the Pearson sample correlation of each matched OTU.
