# Supplementary material for: Neonatal gut and respiratory microbiota: coordinated development through time and space
Source: Microbiome. 2018 Oct 26;6:193. doi: 10.1186/s40168-018-0566-5 (PMC6204011; doi:10.1186/s40168-018-0566-5)
Supplement: Supplementary file 14 — Figure S10. Canonical correlations between body sites. 10-fold crossvalidation allowed unbiased evaluation of the correlation (y-axis) in the first ten subspaces (xaxis) in held-out data (red) and the training data (blue). The correlation is attenuated after adjusting for PMA with a 14 and 25 degree-of-freedom natural spline. The 2 times the standard error of the mean of the cross-validation shows the sampling variability of the correlations. (PDF 43 kb) [file 40168_2018_566_MOESM14_ESM.pdf]

Supplemental Figure 10

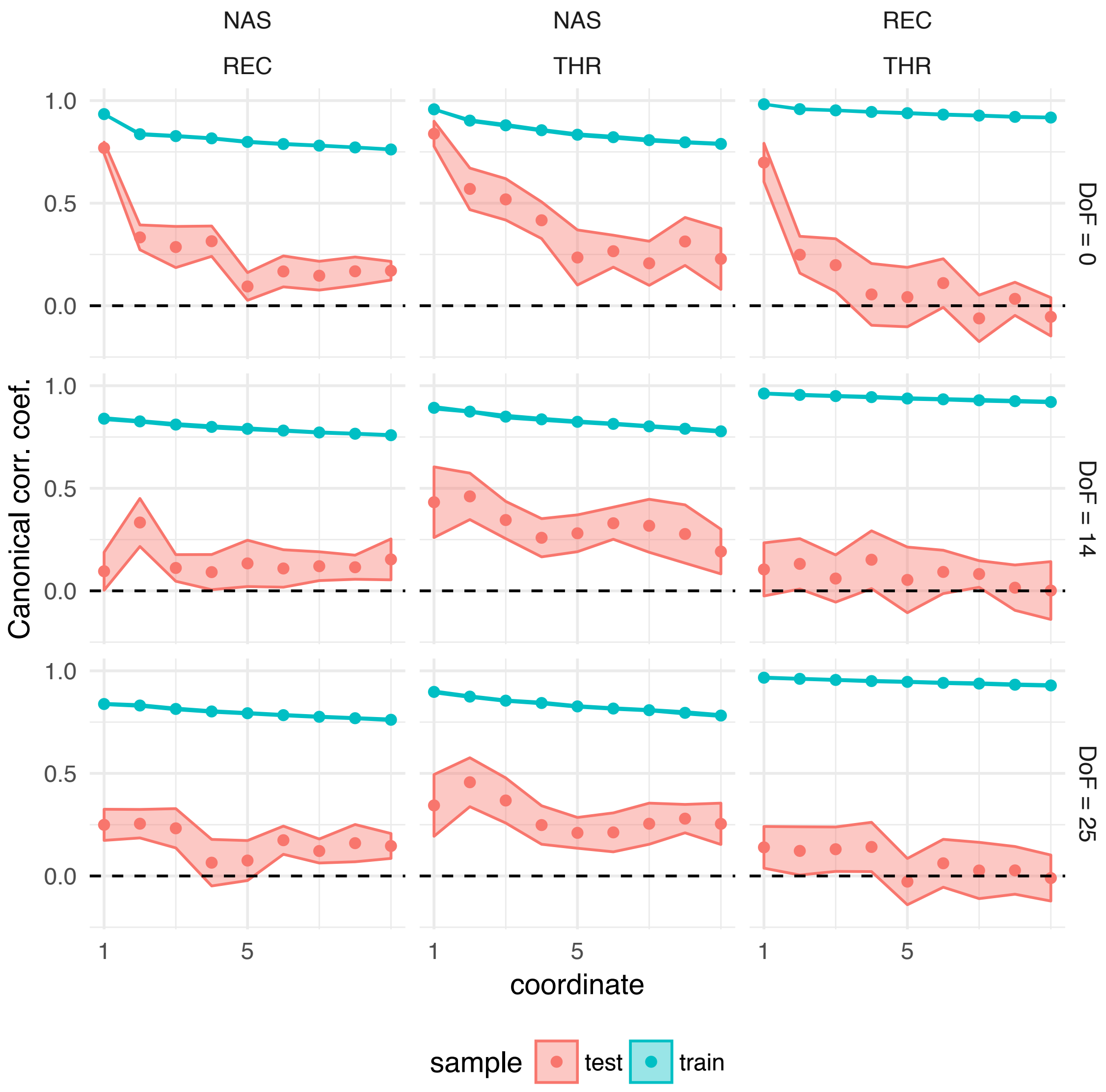

Supplemental Figure 10. Canonical correlations between body sites. 10-fold cross-validation allowed unbiased evaluation of the correlation (y-axis) in the first ten subspaces (x-axis) in held-out data (red) and the training data (blue). The correlation is attenuated after adjusting for PMA with a 14 and 25 degree-of-freedom natural spline. The 2 times the standard error of the mean of the cross-validation shows the sampling variability of the correlations.
